# MAK‐2 Kinase Is Required for Extended Longevity and Enhanced Stress Resistance Resulting From Mild Impairment of Mitochondrial Function in *isp‐1* Mutants

**DOI:** 10.1111/acel.70537

**Published:** 2026-05-13

**Authors:** Ulrich Anglas, Abdelrahman Alokda, Shusen Zhu, Aura A. Tamez González, Ekin Celtikcioglu, Jiaxi Guan, Maisha M. Promi, Grant F. Booth, Alain Pacis, Jeremy M. Van Raamsdonk

**Affiliations:** ^1^ Department of Neurology and Neurosurgery McGill University Montreal Quebec Canada; ^2^ Metabolic Disorders and Complications Program Research Institute of the McGill University Health Centre Montreal Quebec Canada; ^3^ Brain Repair and Integrative Neuroscience Program Research Institute of the McGill University Health Centre Montreal Quebec Canada; ^4^ McGill Genome Centre Montreal Quebec Canada; ^5^ Canadian Centre for Computational Genomics (C3G) McGill University Montreal Quebec Canada; ^6^ Division of Experimental Medicine, Department of Medicine McGill University Montreal Quebec Canada

**Keywords:** aging, *C. elegans*, genetics, kinase, lifespan, mitochondria, reactive oxygen species, signaling

## Abstract

Although mitochondrial function is essential for life in multicellular organisms, work from multiple model organisms has demonstrated that a mild impairment of mitochondrial function can increase lifespan. In *C. elegans* , a mutation in the *isp‐1* gene, which encodes the Rieske iron sulfur protein in Complex III of the mitochondrial electron transport chain, results in increased lifespan, enhanced resistance to stress and slow physiologic rates. While the molecular mechanisms involved are incompletely understood, our working model is that altered mitochondrial function and increased levels of reactive oxygen species lead to changes in nuclear gene expression, including the upregulation of cellular resilience pathways, through mitochondria‐to‐nucleus signaling. In this work, we examine the role of the kinase MAK‐2 and other kinase signaling pathways in the lifespan and stress resistance of *isp‐1* worms. We find that disruption of *mak‐2* specifically decreases the lifespan and stress resistance of *isp‐1* mutants while having no effect on wild‐type animals. Interestingly, kinases from a parallel signaling pathway (MLK‐1/MEK‐1/KGB‐1) are also required for the long‐lifespan of *isp‐1* worms. Finally, we used RNA sequencing to define the role of MAK‐2 in mediating the gene expression changes in *isp‐1* worms. We found that many of the genes that are significantly modulated in *isp‐1* worms are dependent on MAK‐2 including genes involved in innate immunity and stress response. Overall, this work demonstrates an important role for kinase signaling in mediating the lifespan extension and enhanced stress resistance resulting from the mild impairment of mitochondrial function.

## Graphical abstract

Lifespan extension resulting from the mild impairment of mitochondrial function in long‐lived *isp‐1* mutants is dependent on multiple kinase signaling pathways involved in mitochondria‐to‐nucleus communication. These pathways include the MAK‐2 kinase as well as a MLK‐1/MEK‐1/KGB‐1 kinase signaling pathway.

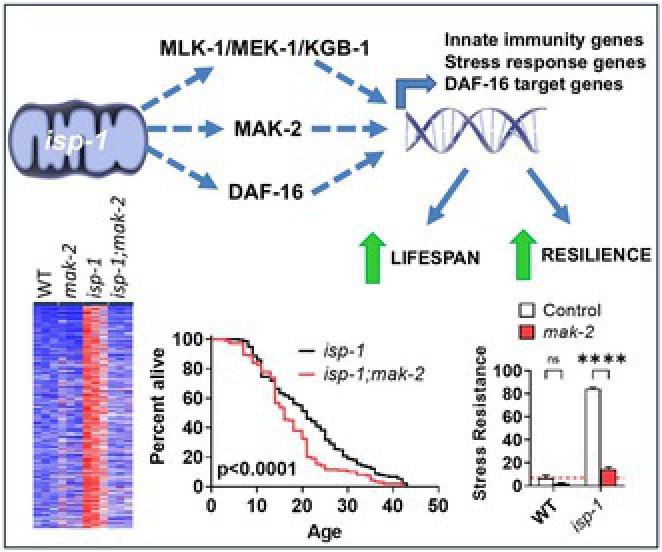

## 1 Introduction

While complete loss of mitochondrial function is often lethal, mild impairment of mitochondrial function can extend longevity. This has been most extensively studied in the genetic model organism *C. elegans* , where multiple mutations that affect mitochondrial function have been shown to increase lifespan. This includes *clk‐1* (Lakowski and Hekimi 1996; Wong et al. 1995), *isp‐1* (Feng et al. 2001), *nuo‐6* (Yang and Hekimi 2010b) and *sod‐2* (Van Raamsdonk and Hekimi 2009), which encode a hydroxylase involved in ubiquinone synthesis, the Rieske iron sulfur protein subunit of Complex III of the mitochondrial electron transport chain, a subunit of Complex I of the mitochondrial electron transport chain, and mitochondrial superoxide dismutase, respectively. RNA interference (RNAi) targeting mitochondrial genes has also been shown to extend longevity (Dillin et al. 2002; Durieux et al. 2011; Lee et al. 2003) with the effect on lifespan being dependent on the level of knockdown (Rea et al. 2007).

Importantly, the ability of mild mitochondrial impairment to increase lifespan is not limited to *C. elegans*. In *Drosophila*, RNAi of genes encoding specific subunits of the mitochondrial electron transport chain complexes I, III, IV and V increases lifespan (Copeland et al. 2009). In mice, disruption of SURF1, which is involved in the assembly of cytochrome c oxidase, results in increased lifespan (Dell'agnello et al. 2007). Also, in mice, the heterozygous deletion of *Mclk‐1*, the mouse homolog of *clk‐1*, extends longevity (Liu et al. 2005).

In exploring the molecular mechanisms involved in lifespan extension caused by mild impairment of mitochondrial function, it has been shown that long‐lived mitochondrial mutants have elevated levels of reactive oxygen species (ROS), which are required for their longevity (Van Raamsdonk and Hekimi 2012; Yang and Hekimi 2010a). The long‐lived mitochondrial mutants exhibit widespread changes in nuclear gene expression (Dues et al. 2017; Ko and Van Raamsdonk 2023; Senchuk et al. 2018; Yee et al. 2014) supporting a role for a mitochondria‐to‐nucleus signaling pathway in their lifespan extension. Among the factors shown to be required for the lifespan of long‐lived mitochondrial mutants include multiple of pathways of cellular resilience including the mitochondrial unfolded protein response (Wu et al. 2018), the p38‐mediated innate immune signaling pathway (Campos et al. 2021), the DAF‐16‐mediated stress response pathway (Senchuk et al. 2018), and the mitochondrial thioredoxin system (Harris‐Gauthier et al. 2022).

A number of other factors have been shown to be required for lifespan extension in long‐lived mitochondrial mutants including the intrinsic apoptosis pathway (Yee et al. 2014), the CEH‐23 homeobox transcription factor (Walter et al. 2011), the p53 homolog CEP‐1 (Baruah et al. 2014), the energy sensing kinase AMPK (Hwang et al. 2014), the hypoxia transcription factor HIF‐1 (S. J. Lee et al. 2010), the SEK‐3 kinase (Munkacsy et al. 2016), the TAF‐4 transcription factor (Khan et al. 2013), and the Golgi protein MON‐2 (Jung et al. 2021). At present it is unclear how these different factors act together to mediate extended longevity.

An important, unanswered question is how perturbations in the mitochondria are communicated to the nucleus to change gene expression. We recently completed a targeted RNAi screen to identify kinases that are required for the long lifespan of *sod‐2* mitochondrial superoxide dismutase mutants (Anglas et al. 2025). Among the kinases identified was MAK‐2, which participates in an established kinase signaling pathway that promotes axonal regeneration (El Bejjani and Hammarlund 2012; Hisamoto and Matsumoto 2017; Pastuhov et al. 2015; Tedeschi and Bradke 2013).

In this work, we explore the role of MAK‐2 in the long lifespan, enhanced stress resistance, and altered physiologic rates of *isp‐1* mitochondrial mutants. We find that disruption of *mak‐2* affects all of these phenotypes in *isp‐1* mutants while having little or no effect in wild‐type animals, suggesting that MAK‐2 is specifically contributing to these phenotypes in *isp‐1* worms. Moreover, we find that multiple other genes from a parallel kinase signaling pathway are also required for the long lifespan of *isp‐1* worms. Finally, we use RNA sequencing (RNA‐seq) to identify differentially expressed genes and pathways in *isp‐1* worms that are dependent on MAK‐2 signaling.

## 2 Results

### 2.1 Loss of *mak‐2* Reduces the Long Lifespan of *isp‐1* Mutants

We recently showed that the kinase MAK‐2 contributes to the extended lifespan of *sod‐2* mitochondrial superoxide dismutase mutants but is dispensable for wild‐type longevity (Anglas et al. 2025). To determine the extent to which MAK‐2 also contributes to the longevity of other long‐lived mutants, we treated a panel of long‐lived genetic mutants with RNAi targeting *mak‐2* and measured lifespan. *mak‐2* RNAi treatment was initiated when worms were pre‐fertile young adults. We found that *mak‐2* RNAi significantly decreased the lifespan of the mitochondrial mutant *isp‐1* but did not reduce the longevity of other long‐lived mutants including *ife‐2, daf‐2, nuo‐6, clk‐1, eat‐2, osm‐5* or *glp‐1* (Figure S1). There was, however, a trend toward decreased lifespan in *daf‐2* and *ife‐2* mutants treated with *mak‐2* RNAi. It is possible that *mak‐2* RNAi would decrease the lifespan of additional long‐lived mutants if RNAi was administered from egg or earlier. It is also interesting to note that *mak‐2* RNAI significantly increased the lifespan of *glp‐1* mutants and resulted in a trend toward increased lifespan in *osm‐5* worms (Figure S1). These results suggest that MAK‐2 may be playing different roles with respect to longevity in different long‐lived mutants and supports the idea that the same pathway can be modulated in different directions to achieve extended longevity through different strategies.

**FIGURE 1:**
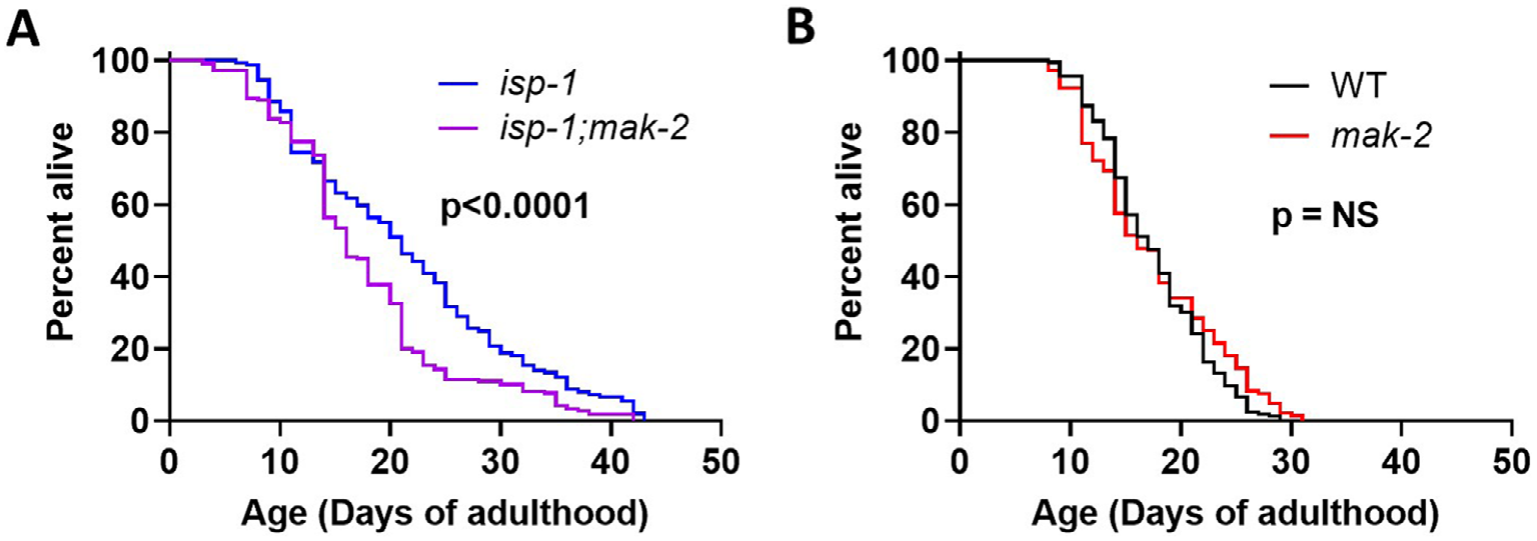
*mak‐2* is specifically required for lifespan extension in *isp‐1* mutants. Loss of *mak‐2* significantly decreased the long‐lifespan of *isp‐1* mutants (A). In contrast, *mak‐2* deletion mutants live as long as wild‐type worms (B). This indicates that the effect of *mak‐2* disruption on lifespan is specific to *isp‐1* worms. Four biological replicates were performed. Statistical significance was assessed using a log‐rank test. Raw lifespan data can be found in Table S1.

In order to confirm the results of the RNAi experiment, we examined the effect of *mak‐2* deletion on *isp‐1* lifespan. To do this, we crossed *isp‐1* worms to *mak‐2(gk1110)* mutants to generate *isp‐1;mak‐2* double mutants and measured lifespan. The *gk1110* mutation is a 1024 bp deletion with a 1 bp insertion that removes exon 2 of the *mak‐2* gene. We found that deletion of *mak‐2* significantly decreased the long lifespan of *isp‐1* mutants but did not affect the longevity of wild‐type worms (Figure 1A,B). This confirms the result of the *mak‐2* RNAi experiment and indicates that *mak‐2* is specifically required for *isp‐1* longevity.

To determine the extent to which MAK‐2 is specifically required for the longevity of *isp‐1* mutants, we examined the extent to which *mak‐2* is needed for the lifespan extension resulting from RNAi knockdown of other components of the mitochondrial electron transport chain that have previously been shown to increase lifespan (Dillin et al. 2002). We treated wild‐type and *mak‐2* mutants with RNAi targeting *nuo‐2* (complex I), *cyc‐1* (complex III), or *cco‐1* (complex IV) and measured lifespan. In each case, we found that RNAi targeting genes encoding subunits of the mitochondrial electron transport chain increased lifespan in wild‐type worms (Figure S2). RNAi treatment in *mak‐2* mutants also resulted in increased lifespan, indicating that *mak‐2* is dispensable for lifespan extension by RNAi targeting mitochondrial genes. This finding provides additional support for previous work showing that genetic mutations affecting the mitochondrial electron transport chain increase lifespan through a different mechanism than RNAi knockdown of mitochondrial genes. For example, previous work by the Hekimi lab showed that the mechanisms contributing to lifespan extension in *isp‐1* mutants are different from *isp‐1* RNAi (Yang and Hekimi 2010b).

### 2.2 Loss of *mak‐2* Decreases the Enhanced Resilience of *isp‐1* Mutants

**FIGURE 2:**
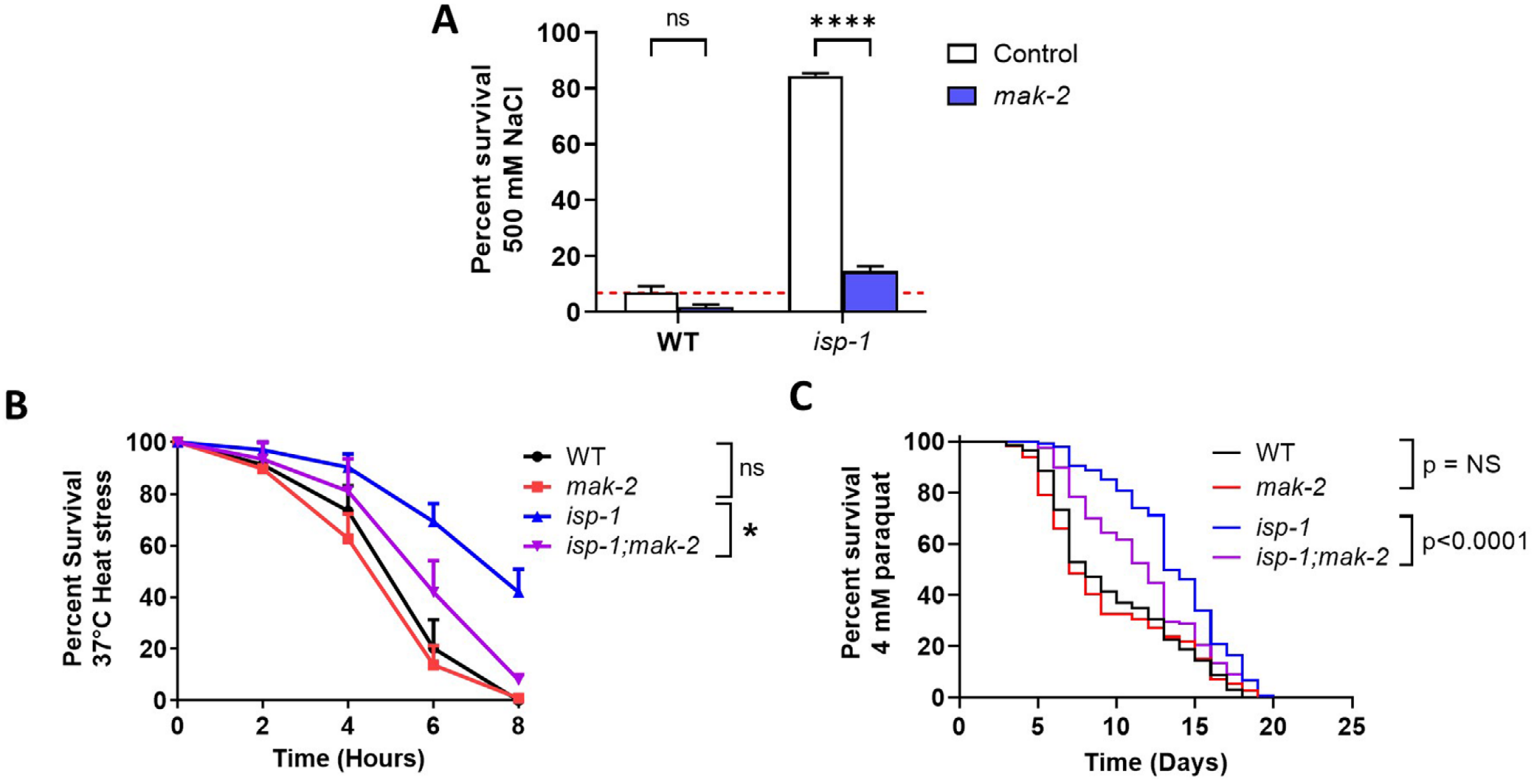
Disruption of *mak‐2* reduces resistance to exogenous stressors specifically in *isp‐1* mutants. Disruption of *mak‐2* significantly decreases the enhanced resistance to osmotic stress (500 mM NaCl; (A)), heat stress (37°C; (B)), and chronic oxidative stress (4 mM paraquat; (C)) in *isp‐1* worms but has no effect on resistance to stress in wild‐type worms. In each case, *isp‐1;mak‐2* worms exhibit increased survival compared to *mak‐2* worms indicating that only part of the enhanced resistance to stress in *isp‐1* worms is MAK‐2 dependent. Three biological replicates were performed. Statistical significance was assessed using a two‐way ANOVA with Šidák's multiple comparisons test in panel A, a two‐way repeated measures ANOVA with Tukey's multiple comparisons test in panel B, and a log‐rank test in panel C. Error bars indicate SEM. *p < 0.05, ****p < 0.0001.

To determine whether *mak‐2* is also required for the enhanced resilience of *isp‐1* worms, we compared resistance to exogenous stressors between *isp‐1* and *isp‐1;mak‐2* worms. We and others have previously shown that *isp‐1* worms have increased resistance to osmotic stress, heat stress, and chronic oxidative stress (Dues et al. 2017; Harris‐Gauthier et al. 2022). In examining resistance to osmotic stress, we found that *isp‐1* worms exhibit increased survival, which is markedly decreased by the absence of *mak‐2* (Figure 2A; 500 mM NaCl). In contrast, *mak‐2* deletion did not affect osmotic stress resistance in wild‐type worms. Similarly, *isp‐1* mutants showed increased resistance to heat stress (Figure 2B; 37°C) and oxidative stress (Figure 2C; 4 mM paraquat). In each case, disruption of *mak‐2* significantly decreased resistance to stress in *isp‐1* worms but had no effect on wild‐type survival. Combined, this demonstrates that *mak‐2* is specifically required for the enhanced stress resistance of *isp‐1* worms. It should also be noted that for all three exogenous stressors, *isp‐1;mak‐2* worms showed increased survival compared to *mak‐2* worms (Figure 2A–C). This indicates that part of the enhanced stress resistance of *isp‐1* worms is independent of MAK‐2.

### 2.3 Loss of *mak‐2* Further Slows Physiologic Rates in *isp‐1* Mutants

**FIGURE 3:**
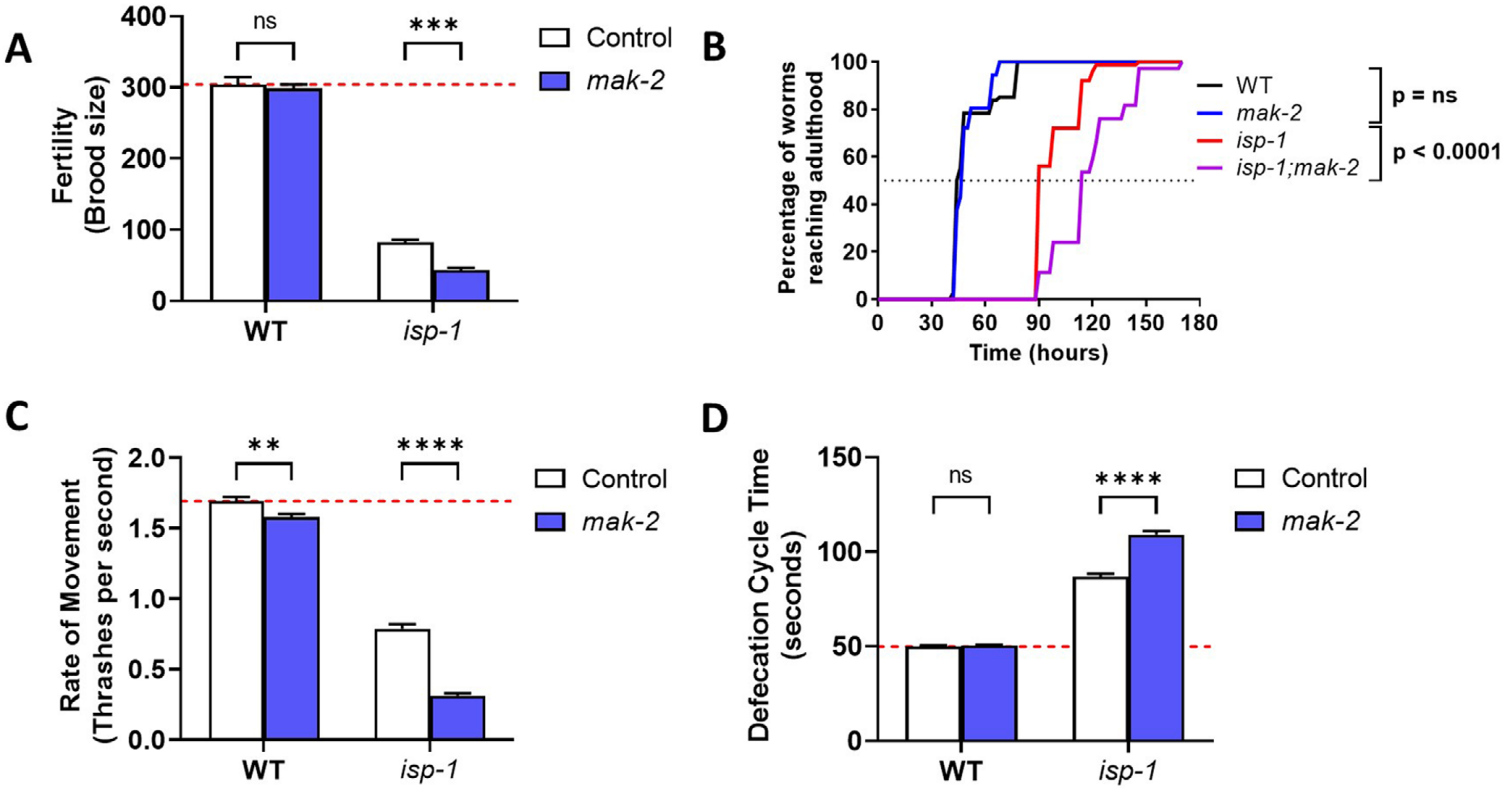
Disruption of *mak‐2* exacerbates slowing of physiologic rates in *isp‐1* mutants. (A) Disruption of *mak‐2* further decreases the reduced brood size of *isp‐1* worms but does not affect wild‐type fertility. (B) Deletion of *mak‐2* significantly slowed the development of *isp‐1* worms but did not affect the development of wild‐type worms. (C) Disruption of *mak‐2* resulted in a small decrease in the thrashing rate in wild‐type worms while markedly reducing movement in *isp‐1* mutants. (D) Loss of *mak‐2* further slowed the slow defecation cycle length in *isp‐1* mutants but did not affect defecation cycle length in wild‐type worms. Three biological replicates were performed. Statistical significance was assessed using a two‐way ANOVA with Šidák's multiple comparisons test in panels A, C and D, and a log‐rank test in panel B. Error bars indicate SEM. **p < 0.01, ***p < 0.001, ****p < 0.0001.

Having shown that *mak‐2* is needed for the enhanced lifespan and stress resistance of *isp‐1* worms, we next sought to determine the role of *mak‐2* in mediating the slow physiologic rates of *isp‐1* worms. While *isp‐1* worms have decreased fertility (brood size) compared to wild‐type worms, this deficit is further exacerbated by disruption of *mak‐2* (Figure 3A). Deletion of *mak‐2* also further slowed the already lengthened post‐embryonic development time of *isp‐1* worms (Figure 3B). Similarly, *isp‐1* worms have a decreased rate of movement compared to wild‐type worms, and their movement is further decreased when *mak‐2* is disrupted (Figure 3C). Finally, *isp‐1* worms exhibit a markedly slowed defecation cycle length that is further slowed when *mak‐2* is deleted (Figure 3D). In contrast to the effect of *mak‐2* deletion on *isp‐1* worms, disruption of *mak‐2* in wild‐type animals did not affect fertility, development, or defecation rate and only had a small effect on mobility (Figure 3A–D). This shows that the effect of *mak‐2* on physiologic rates is mostly specific to *isp‐1* mutants.

Previous studies have shown that *isp‐1* worms exhibit elevated levels of reactive oxygen species and activation of the mitochondrial unfolded protein response (mitoUPR), both of which are required for these worms to live long (Van Raamsdonk and Hekimi 2012; Wu et al. 2018; Yang and Hekimi 2010a). As disruption of *mak‐2* decreases *isp‐1* lifespan, we sought to determine the extent to which this disruption affects ROS levels and mitoUPR activation. To measure ROS levels, we stained *isp‐1* and *isp‐1;mak‐2* worms with the ROS‐sensitive dye dihydroethidium (DHE), which we and others have previously shown to exhibit increased staining in *isp‐1* worms compared to wild‐type animals (Dues et al. 2017; Lee et al. 2010). We did not observe any difference in DHE staining between *isp‐1* and *isp‐1;mak‐2* worms, suggesting that deletion of *mak‐2* does not alter ROS levels in *isp‐1* mutants (Figure S3).

Next, we examined the role of *mak‐2* in mitoUPR activation. To do this, we compared the expression levels of ATFS‐1 target genes in *isp‐1* and *isp‐1;mak‐2* worms (Soo and Van Raamsdonk 2021). As we have previously reported, the majority of the high confidence ATFS‐1 target genes were found to be upregulated in *isp‐1* worms (Figure S4). The ATFS‐1 target genes were also found to be upregulated in *isp‐1;mak‐2* worms. The fact that the *mak‐2* deletion does not decrease the expression of ATFS‐1 target genes in *isp‐1* worms indicates that MAK‐2 is not involved in mitoUPR activation in *isp‐1* mutants.

### 2.4 MAK‐2 Mediates Gene Expression Changes in *isp‐1* Mutants

Since disruption of *mak‐2* specifically affected the lifespan, stress resistance, and physiologic rates of *isp‐1* worms, we next examined the effect of *mak‐2* on *isp‐1* gene expression to gain insight into the molecular mechanisms involved. To do this, we used RNA‐seq to compare gene expression between *isp‐1* and *isp‐1;mak‐2* worms across the entire genome in an unbiased manner with wild‐type and *mak‐2* worms as controls. A principal component analysis (PCA) revealed that the three biological replicates from the four different genotypes (*isp‐1, isp‐1;mak‐2*, wild‐type, *mak‐2*) cluster closely together and away from other genotypes (Figure S5A). Consistent with our phenotyping results, this plot indicates that the gene expression changes resulting from *mak‐2* deletion are greater in *isp‐1* worms than in a wild‐type background.

**FIGURE 4:**
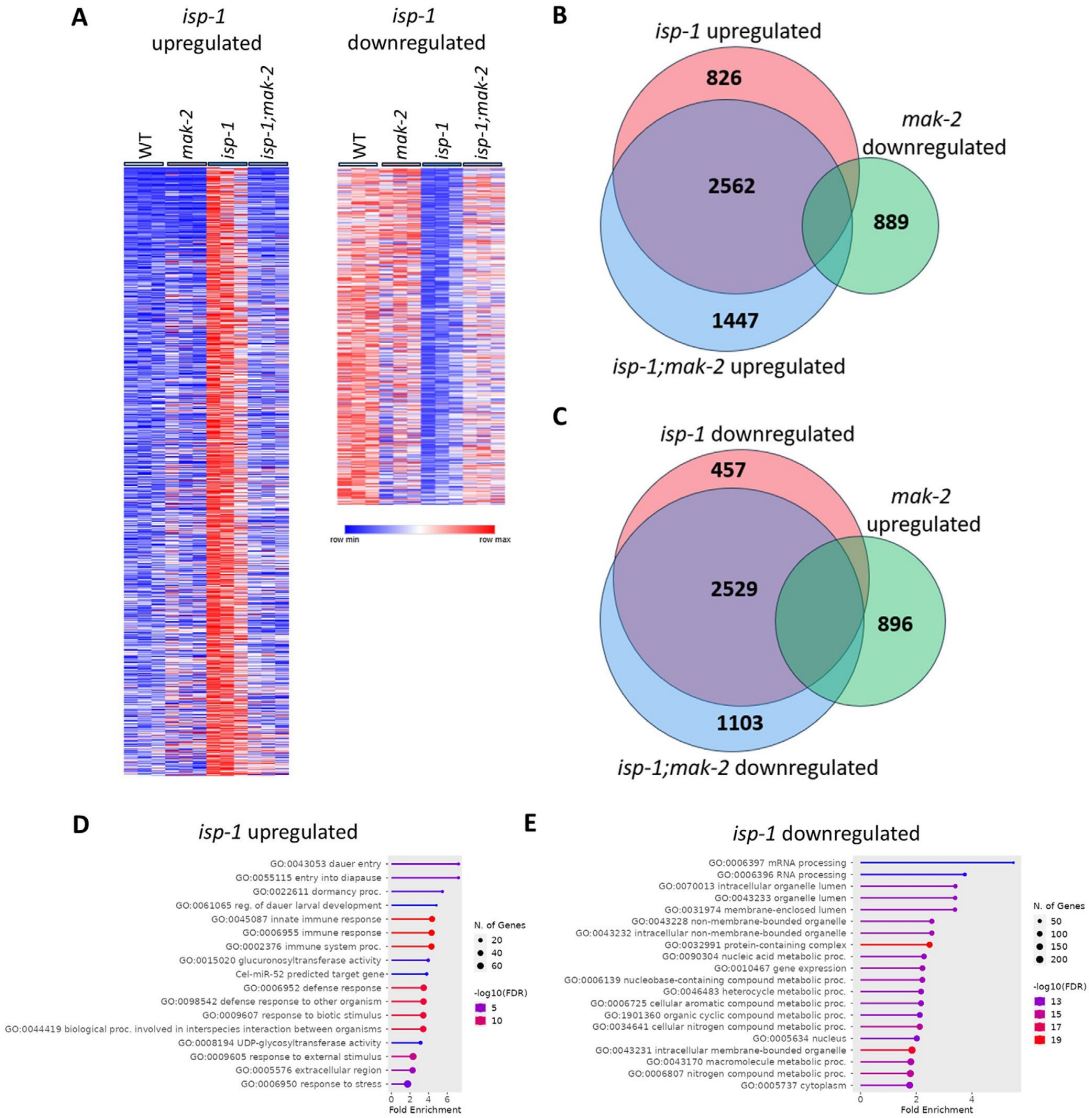
MAK‐2 is required for transcriptional changes in *isp‐1* mutants. RNA sequencing was used to compare gene expression between *isp‐1* mutants and *isp‐1* mutants in which *mak‐2* is deleted. (A) Heat maps showing genes that are upregulated and downregulated in *isp‐1* mutants that are dependent on *mak‐2*. (B) Venn diagram showing overlap between upregulated genes in *isp‐1* and *isp‐1;mak‐2* worms with genes downregulated in *mak‐2* mutants. (C) Venn diagram showing overlap between downregulated genes in *isp‐1* and *isp‐1;mak‐2* worms with genes upregulated in *mak‐2* mutants. (D) Gene ontology (GO) enrichment analysis for 826 genes upregulated in *isp‐1* worms that are dependent on *mak‐2*. (E) GO enrichment analysis for 457 genes downregulated in *isp‐1* worms that are dependent on *mak‐2*.

Comparison of gene expression across the four genotypes reveals that there are a number of genes that are significantly upregulated or downregulated in *isp‐1* worms that are reverted to wild‐type by the deletion of *mak‐2* (Figure 4A). This indicates that *mak‐2* is required for many of the transcriptional changes that are present in *isp‐1* mutants. There are also a large number of differentially expressed genes in *isp‐1* worms that are *mak‐2*‐independent such that both *isp‐1* and *isp‐1;mak‐2* worms have a large number of differentially expressed genes compared to wild‐type worms (*isp‐1*: 3669 upregulated genes, *isp‐1;mak‐2*: 4372 upregulated genes) (Figure S5B,C). Disruption of *mak‐2* in a wild‐type background also resulted in widespread changes in gene expression (1806 upregulated genes, 1320 downregulated genes) (Figure S6).

To gain insight into the function of genes that are differentially expressed in *isp‐1* worms in a *mak‐2‐*dependent manner, we identified genes that are (1) upregulated in *isp‐1* worms, (2) not upregulated in *isp‐1;mak‐2* worms, and (3) not downregulated in *mak‐2* worms (Figure 4B). This analysis yielded 826 genes, which were then used for enrichment analysis (See Table S2 for a list of these genes). Similarly, we identified genes that are (1) downregulated in *isp‐1* worms, (2) not downregulated in *isp‐1;mak‐2* worms, and (3) not upregulated in *mak‐2* worms (Figure 4C). This resulted in a list of 457 genes, which were similarly used for enrichment analysis (See Table S2 for a list of these genes). We found that genes that are upregulated in *isp‐1* worms in a *mak‐2‐*dependent manner were enriched for genes involved in innate immune response, response to stress, and dauer/diapause entry (Figure 4D; Figure S7). All of these functions have been previously associated with longevity. Genes that are downregulated in *isp‐1* worms in a *mak‐2‐*dependent manner were enriched for genes involved in various metabolic processes, RNA processing, and gene expression (Figure 4E; Figure S8).

In order to gain further insight into the differentially expressed genes in *isp‐1* worms that are dependent on MAK‐2, we performed transcription factor analyses. We used two complementary approaches. First, we used the CeIEsT Shiny app (https://doi.org/10.1093/genetics/iyae189). This app integrates existing ChIP‐seq and DNA‐binding motif information and then uses a univariate linear model (ULM) or multivariate linear model (MLM) to predict transcription factors that would bind to the gene set entered. Second, we did motif enrichment analysis. This analysis examines the promoter regions of the genes in the gene set and asks which transcription factor binding motifs are enriched in these promoter regions. Using these analyses, we identified multiple transcription factors predicted to be involved in the upregulated and downregulated gene sets (Table S3). Of note, there were three transcription factors, *jun‐1, end‐3*, and *efl‐2*, that were identified by both approaches. In future research, it would be interesting to examine if and how these transcription factors are activated by MAK‐2 as well as the extent to which they are required for *isp‐1* longevity.

To determine the role of elevated ROS in the differential expression of genes in *isp‐1* worms, we compared genes that are upregulated and downregulated in *isp‐1* worms to genes that are modulated by exposure to the ROS‐generating compound paraquat. When comparing all of the genes that are differentially expressed in *isp‐1* worms to genes that are differentially expressed after exposure to a lifespan‐extending dose of 0.1 mM paraquat (Yang and Hekimi 2010a), we found that there was a statistically significant overlap between the two gene sets (Figure S9B). In contrast, when we specifically compared genes that are differentially expressed in *isp‐1* worms in a MAK‐2‐dependent manner, we found that the percentage overlap is significantly less than would be expected by chance (Figure S9C). This suggests that while elevated ROS contributes to many of the changes in gene expression in *isp‐1* worms, the subset of transcriptomic changes that are dependent on MAK‐2 are not dependent on ROS.

### 2.5 MLK‐1/MEK‐1/KGB‐1 Signaling Pathway Is Required for Extended Longevity of *isp‐1* Mutants

Previous studies have shown that MAK‐2 is involved in kinase signaling pathways that promote axon regeneration. MAK‐2 acts in a pathway involving the kinases DLK‐1, MKK‐4, and PMK‐3, which act upstream of MAK‐2, and the CEBP‐1 transcription factor, which acts downstream of MAK‐2 (DLK‐1 ➔ MKK‐4 ➔ PMK‐3 ➔ MAK‐2 ➔ CEBP‐1) (El Bejjani and Hammarlund 2012; Hisamoto and Matsumoto 2017; Pastuhov et al. 2015; Tedeschi and Bradke 2013). Another pathway implicated in axon regeneration involves the kinases MLK‐1, MEK‐1, KGB‐1, and the transcription factor FOS‐1 (MLK‐1 ➔ MEK‐1 ➔ KGB‐1 ➔ FOS‐1) (El Bejjani and Hammarlund 2012; Hisamoto and Matsumoto 2017; Pastuhov et al. 2015; Tedeschi and Bradke 2013). In addition, there is considerable crosstalk between the two pathways. For example, the E3 ubiquitin ligase RPM‐1 can inhibit both pathways by targeting DLK‐1 and MLK‐1 for ubiquitin‐mediated degradation (El Bejjani and Hammarlund 2012; Nakata et al. 2005; Nix et al. 2011; Pastuhov et al. 2015). Similarly, the phosphatase VHP‐1 can inhibit both pathways by inactivating PMK‐3 or KGB‐1 (El Bejjani and Hammarlund 2012; Nix et al. 2011; Pastuhov et al. 2015). The phosphatases PPM‐1 and PPM‐2 have also been shown to inhibit both the MLK‐1 and DLK‐1 pathways (Baker et al. 2014; Tulgren et al. 2011). To assess the role of other proteins in these axon regeneration pathways in the longevity of *isp‐1* worms, we knocked down the expression of the genes encoding each of these proteins using RNAi and measured the resulting effect on lifespan.

As a first step, we examined the expression levels of each of these genes in the RNA‐seq data. We found that the levels of *mak‐2* in *isp‐1* mutants are equivalent to wild‐type (Figure S10). Similarly, there was no change in the expression of *mkk‐4, mlk‐1, mek‐1, kgb‐1*, or *rpm‐1* in *isp‐1* worms. There was a small increase in the levels of *dlk‐1, pmk‐3, cebp‐1, fos‐1*, and *vhp‐1* in *isp‐1* worms.

**FIGURE 5:**
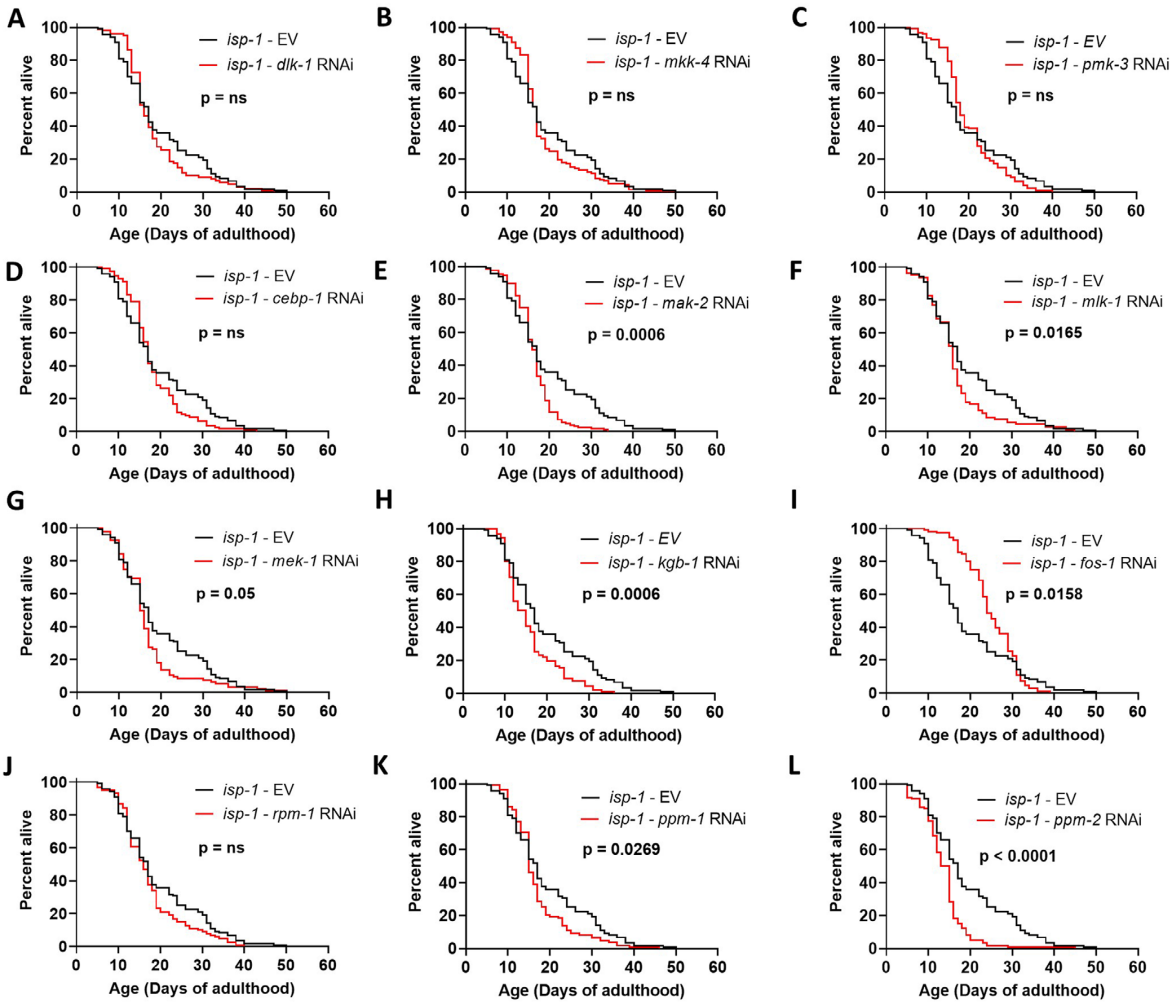
MLK‐1/MEK‐1/KGB‐1 kinase signaling pathway is required for the extended longevity of *isp‐1* mutants. RNA interference was used to knock down the expression of other kinases involved in axon regeneration pathways in *isp‐1* worms and the resulting effect on lifespan was quantified. Knocking down *dlk‐1* (A), *mkk‐4* (B), *pmk‐3* (C), or *cebp‐1* (D) in the DLK‐1 pathway did not affect the lifespan of *isp‐1* worms, despite *mak‐2* being required for *isp‐1* longevity (E). In contrast, disruption of *mlk‐1* (F), *mek‐1* (G), and *kgb‐1* (H), all decreased *isp‐1* lifespan. RNAi against *fos‐1* increased mean lifespan while decreasing maximum lifespan in *isp‐1* worms (I). Disruption of *rpm‐1* did not affect *isp‐1* lifespan, while RNAi against either of the phosphatase genes *ppm‐1* or *ppm‐2* decreased *isp‐1* lifespan (K, L). Three biological replicates were performed. Statistical significance was assessed using a log‐rank test. Raw lifespan data can be found in Table S1.

In examining the effect of the axon regeneration pathway genes on *isp‐1* lifespan, we found that although *mak‐2* RNAi decreases *isp‐1* lifespan, decreasing the expression of other components of the DLK‐1 pathway, including *dlk‐1*, *mkk‐2*, *pmk‐3*, and *cebp‐1*, did not affect the longevity of *isp‐1* worms (Figure 5A–E). In contrast, RNAi targeting genes involved in the MLK‐1 pathway decreased *isp‐1* lifespan, including *mlk‐1, mek‐1*, and *kgb‐1* (Figure 5F–H). RNAi against *fos‐1* increased mean lifespan while decreasing the maximum lifespan of *isp‐1* worms (Figure 5I). Disruption of the E3 ubiquitin ligase gene *rpm‐1*, or the phosphatase‐encoding genes *ppm‐1* or *ppm‐2*, all decreased *isp‐1* longevity (Figure 5J–L). Finally, *isp‐1* worms failed to develop to adulthood on RNAi bacteria targeting the *vhp‐1* phosphatase gene.

Having shown that both MAK‐2 and the MLK‐1/MEK‐1/KGB‐1 pathway are required for *isp‐1* longevity, we wondered if these two pathways are acting together to modulate the same genetic targets. To test this idea, we examined the effect of disrupting the MLK‐1/MEK‐1/KGB‐1 pathway on the expression of genes that are upregulated in *isp‐1* worms in a MAK‐2‐dependent manner. We found that using RNAi to knock down the expression of *mlk‐1, mek‐1, kgb‐1*, or *fos‐1* did not significantly decrease the expression of genes that are upregulated in *isp‐1* in a MAK‐2‐dependent manner (Figure S11). This suggests that MAK‐2 and the MLK‐1/MEK‐1/KGB‐1 pathway are modulating different sets of genes to promote longevity in *isp‐1* worms.

### 2.6 Distinct Kinase Signaling Pathways Are Required for Lifespan Extension in Long‐Lived *sod‐2* and *isp‐1* Mutants

**FIGURE 6:**
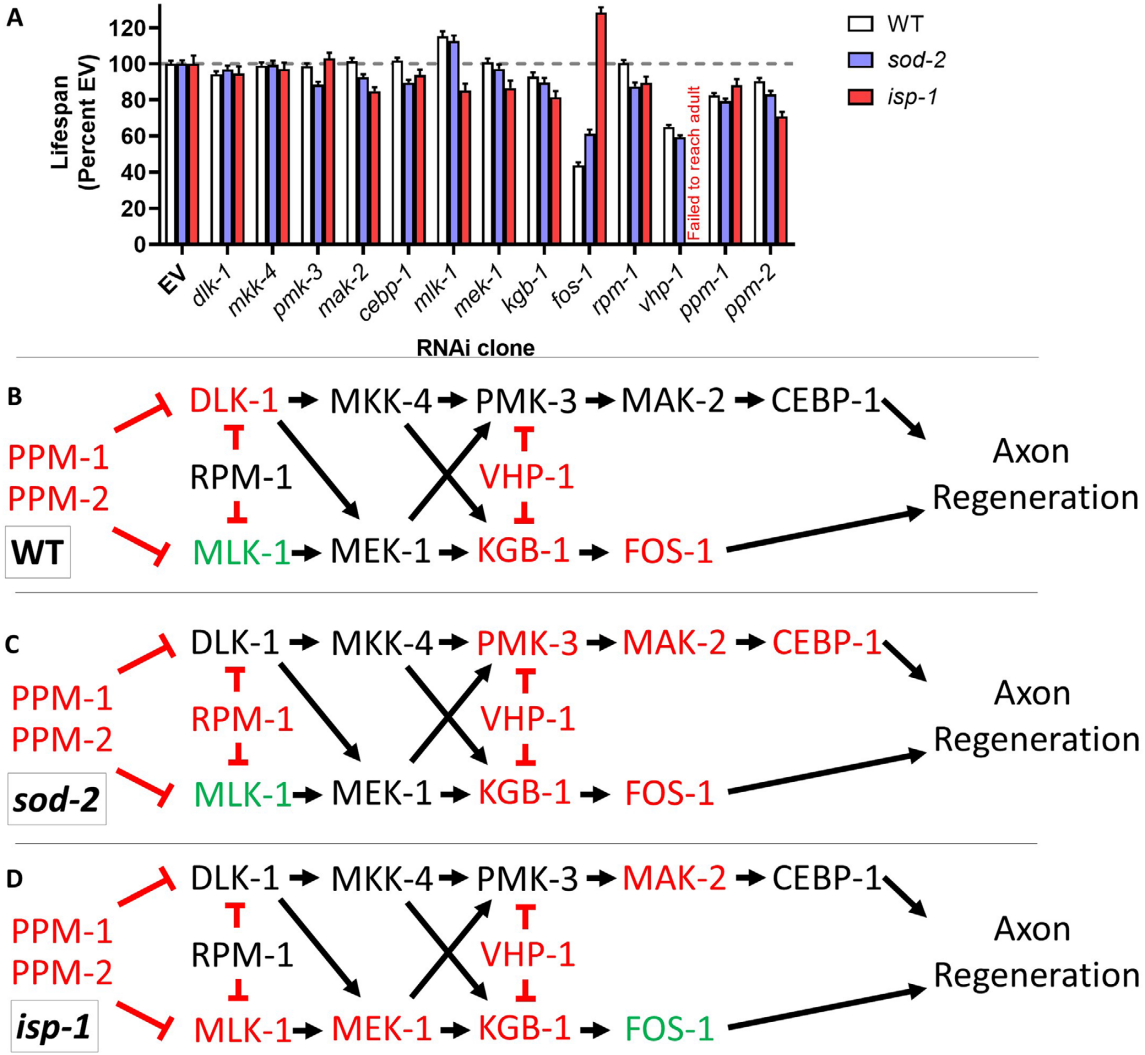
Different kinase signaling pathways are required for wild‐type, *sod‐2* and *isp‐1* lifespan. (A) Bar graph showing mean lifespan of wild‐type, *sod‐2* and *isp‐1* worms treated with RNAi clones targeting components of the DLK‐1 and MLK‐1 axon regeneration pathways. Lifespan is expressed as a percent of empty vector (EV). Gray dotted line indicates 100%. Axon regeneration genes affecting lifespan in wild‐type (B), *sod‐2* (C), and *isp‐1* (D) worms. Disruption of the phosphatase genes *vhp‐1, ppm‐1* or *ppm‐2* reduced lifespan in all three strains as did disruption of *kgb‐1*. In *sod‐2* worms, a PMK‐3‐MAK‐2‐CEBP‐1 pathway was specifically required for their long lifespan. In *isp‐1* worms, a MLK‐1‐MEK‐1‐KGB‐1 pathway was specifically required for their longevity. *mak‐2* was the only gene that was required for the long lifespan of both *sod‐2* and *isp‐1* worms but not wild‐type worms. Data on wild‐type and *sod‐2* mutants is from Anglas et al. 2025. Data on *isp‐1* worms is from Figure 5.

Comparing these results to our previous results from *sod‐2* and wild‐type worms indicates that, while disruption of some kinases is generally detrimental for lifespan, the lifespan of each strain is dependent on a unique set of kinases (Figure 6A). RNAi against the *ppm‐1, ppm‐2*, or *vhp‐1* phosphatase genes decreased lifespan in all three strains, as did RNAi targeting *kgb‐1* (Figure 6A–D). In *sod‐2* worms, a signaling pathway involving SEK‐3/PMK‐3/MAK‐2/CEBP‐1 is specifically required for the long lifespan of these worms but is dispensable for wild‐type lifespan (Figure 6C) (Anglas et al. 2025). With the exception of MAK‐2, the components of this pathway are also dispensable for *isp‐1* lifespan. In contrast, *isp‐1* worms require a MLK‐1/MEK‐1/KGB‐1 signaling pathway for their enhanced longevity, which is not needed for longevity in *sod‐2* or wild‐type worms (Figure 6D). Combined, this indicates that kinase signaling pathways are important for lifespan extension in multiple long‐lived mutants, but different pathways are required in *sod‐2* and *isp‐1* mutants.

In *sod‐2* worms there is a MAK‐2‐dependent upregulation of CEBP‐1 target genes (Anglas et al. 2025). Since MAK‐2 was also found to be required for *isp‐1* longevity, we examined the RNA‐seq data to determine if CEBP‐1 target genes are also upregulated in *isp‐1* worms. Comparing genes that are significantly upregulated in *isp‐1* worms (3669 genes) to genes identified as CEBP‐1 target genes in a previous chromatin immunoprecipitation (ChIP) experiment (212 genes) (Kim et al. 2016), we observed an overlap of 42 genes, which is not significantly different than would be expected if two similarly sized gene sets were chosen at random (Figure S12A). Similarly, comparing genes that are upregulated in *isp‐1* worms in a MAK‐2‐dependent manner (826 genes) to CEBP‐1 target genes, we observed an overlap of 11 genes, which again is not significantly different than would be expected if two similarly sized gene sets were chosen at random (Figure S12B). Of the 42 CEBP‐1 target genes that are upregulated in *isp‐1* worms, almost all of them are still upregulated in *isp‐1* worms when *mak‐2* is deleted (Figure S12C,D), indicating that their upregulation is not dependent on MAK‐2. Combined, these results indicate that there is no enrichment of CEBP‐1 target genes among the genes upregulated in *isp‐1* worms and those CEBP‐1 target genes that are upregulated are not dependent on MAK‐2.

We have previously shown that DAF‐16 target genes are upregulated in *isp‐1* worms in a DAF‐16‐dependent manner and that DAF‐16 is required for the long lifespan of *isp‐1* mutants (Senchuk et al. 2018). As previous work has shown that KGB‐1 can activate DAF‐16 (Twumasi‐Boateng et al. 2012; Uno et al. 2013), our results suggest the possibility that the MLK‐1/MEK‐1/KGB‐1 pathway may be extending lifespan in *isp‐1* worms through activation of DAF‐16. To assess the role of the MLK‐1/MEK‐1/KGB‐1 pathway in activating DAF‐16 target genes, we treated worms with RNAi targeting *mlk‐1, mek‐1, kgb‐1, fos‐1* or *daf‐16* and then measured the expression of six DAF‐16 target genes (*mtl‐1, dod‐3, sodh‐1, ftn‐1, icl‐1*, and *sod‐3*) using quantitative RT‐PCR. As we have previously reported (Senchuk et al. 2018), DAF‐16 target genes are upregulated in *isp‐1* worms (Figure S13). While *daf‐16* RNAi decreased the expression of the DAF‐16 target genes, RNAi targeting components of the MLK‐1/MEK‐1/KGB‐1 pathway had little or no effect on the upregulation of DAF‐16 target genes in *isp‐1* worms (Figure S13). This suggests that DAF‐16 target genes are upregulated in *isp‐1* worms independently of the MLK‐1/MEK‐1/KGB‐1 pathway. Comparing gene expression between *isp‐1* and *isp‐1;mak‐2* worms using our RNA‐seq data demonstrated that the upregulation of DAF‐16 target genes in *isp‐1* worms is also independent of MAK‐2 (Figure S14). Moreover, knockdown of *mak‐2* or genes in the *mlk‐1/mek‐1/kgb‐1* pathway did not increase the nuclear localization of DAF‐16 (Figure S15). In addition, knockdown of *daf‐16* using RNAi was found to similarly decrease the lifespans of *isp‐1* and *isp‐1;mak‐2* (Figure S16) indicating that the detrimental effects of disrupting *mak‐2* or *daf‐16* on *isp‐1* lifespan are not additive.

## 3 Discussion

In this work, we examined the contribution of MAK‐2 to the extended lifespan of long‐lived mutants. We found that MAK‐2 is required for the long‐lifespan and enhanced stress resistance of *isp‐1* worms. Exploration of other genetic pathways involving MAK‐2 identified a MLK‐1/MEK‐1/KGB‐1 signaling pathway that is required for *isp‐1* longevity. Finally, RNA‐seq comparing gene expression in *isp‐1* worms with and without MAK‐2 signaling showed that MAK‐2 is required for the differential expression of a large number of genes in *isp‐1* mutants including those involved in innate immunity and resilience.

### 3.1 MAK‐2 Kinase Contributes to Lifespan Extension and Enhanced Resistance to Stress

Aside from our recent work showing that *mak‐2* is required for lifespan extension in *sod‐2* mitochondrial superoxide dismutase mutants (Anglas et al. 2025), we were only able to identify one other report in which the role of *mak‐2* in longevity was examined. In a paper examining the relationship between nuclear DNA damage checkpoint proteins and impaired mitochondrial function, it was found that knocking down *mak‐2* with RNAi did not shorten the lifespan extension resulting from RNAi targeting *atp‐3*, a gene encoding a subunit of ATP synthase (Borror et al. 2022). As we have reported here and previously, *mak‐2* RNAi also did not decrease wild‐type lifespan (Anglas et al. 2025; Borror et al. 2022). Interestingly, when either of the nuclear DNA checkpoint genes *atl‐1* or *atm‐1* are disrupted individually, but not both together, RNAi against *mak‐2* partially reduces the lifespan extension resulting from *atp‐3* RNAi (Borror et al. 2022). Combined, this suggests that *mak‐2* is only required for lifespan extension under specific conditions but generally not required for normal lifespan.

Our results suggest that multiple long‐lived genetic mutants and worms treated with RNAi targeting mitochondrial electron transport genes do not require MAK‐2 for lifespan extension. In contrast, we have shown that both *isp‐1* and *sod‐2* worms (Anglas et al. 2025) are dependent on MAK‐2 for their extended longevity. It is unclear what is unique about *isp‐1* and *sod‐2* especially compared to the other mitochondrial mutants, *nuo‐6* and *clk‐1*, which have many overlapping changes in gene expression and require many of the same pathways to achieve long life (Campos et al. 2021; Harris‐Gauthier et al. 2022; Senchuk et al. 2018; Wu et al. 2018). As the RNAi targeting *mak‐2* was initiated after development, it is possible that other long‐lived mutants require *mak‐2* for their longevity but no longer need it during adulthood. Future analyses comparing *isp‐1* and *sod‐2* to the other long‐lived mutants tested may provide insights into why MAK‐2 seems to be uniquely required in these two mutants.

In addition to decreasing *isp‐1* lifespan, disruption of *mak‐2* also decreased resistance to multiple exogenous stressors in *isp‐1* worms but not wild‐type worms. We previously observed that *mak‐2* deletion significantly decreases the resistance of *sod‐2* worms to both heat and osmotic stress, but not chronic oxidative stress (Anglas et al. 2025). This may be because resistance to chronic oxidative stress is already decreased in *sod‐2* mutants, while *isp‐1* worms exhibit increased resistance to chronic oxidative stress. In our previous study, we observed a significant effect of *mak‐2* deletion on resistance to osmotic stress in wild‐type worms. In the current study, we observed a trend toward decreased resistance, which failed to reach significance most likely because a higher concentration of NaCl (500 mM) was used in this study compared to the previous study (450 mM) thereby reducing the window to observe a decrease.

In examining the effect of *mak‐2* on the physiologic rates of *isp‐1* worms, we observed a significant exacerbation of the already altered fertility, development time, rate of movement and defecation cycle length, with little or no effect in wild‐type worms. The fact that *mak‐2* deletion reverts *isp‐1* lifespan toward wild‐type while increasing the difference in physiologic rates indicates that these two phenotypes can be experimentally dissociated and suggests that the mechanisms leading to these phenotypes are distinct.

In this work, we found that there are 826 genes upregulated in *isp‐1* worms in a MAK‐2 dependent manner and 452 genes downregulated in *isp‐1* worms in MAK‐2 dependent manner. To gain insight into whether any of these differentially expressed genes are affecting *isp‐1* phenotypes, we reviewed the literature on select modulated genes. We found that multiple of the genes that we identified have previously been shown to affect phenotypes in *isp‐1* worms. For example, *nsy‐1*, *aak‐2, tbb‐6* and *sek‐3* are all upregulated in *isp‐1* worms in a MAK‐2‐dependent manner. Deletion of any of these genes decreases *isp‐1* lifespan (Campos et al. 2021; Curtis et al. 2006; Hwang et al. 2014; Khan et al. 2013). Similarly, *lipl‐4* and *nhr‐8* are both upregulated in *isp‐1* worms in a MAK‐2‐dependent manner. While a role in *isp‐1* lifespan has not been demonstrated, *lipl‐4* was found to increase lifespan in wild‐type worms (Ramachandran et al. 2019) and *nhr‐8* has been implicated in lifespan resulting from inhibition of calcineurin (*tax‐6* RNAi) (Das et al. 2024). Combined, this indicates the multiple genes that are upregulated in *isp‐1* worms in a MAK‐2‐dependent manner can affect lifespan individually.

### 3.2 Role of MLK‐1 ➔ MEK‐1 ➔ KGB‐1 Pathway in Longevity

Since MAK‐2 has an established role in a genetic pathway that mediates axonal regeneration, we examined the role of other proteins involved in axon regeneration in extending the lifespan of *isp‐1* mutants. Unlike *sod‐2* worms, we found that the PMK‐3 kinase that activates MAK‐2 and the CEBP‐1 transcription factor that MAK‐2 activates are not required for the long lifespan of *isp‐1* mutants. Instead, we found that disruption of MLK‐1 or MEK‐1 specifically prevented lifespan extension in *isp‐1* mutants but did not decrease lifespan in wild‐type worms. In fact, deletion of *mlk‐1* extended longevity in wild‐type worms. Disruption of KGB‐1 also decreased *isp‐1* lifespan but decreased wild‐type lifespan as well making this observation harder to interpret.

Although it is possible that the MLK‐1/MEK‐1/KGB‐1 pathway affects *isp‐1* lifespan through an effect on axon regeneration, if this were true then we would expect that disruption of any of the genes in the axon regeneration pathways would decrease *isp‐1* longevity. Since we observed no effect of *dlk‐1, mkk‐4, pmk‐3*, or *cebp‐1* on *isp‐1* lifespan, we think that it is likely an axon regeneration‐independent function of the MLK‐1/MEK‐1/KGB‐1 pathway that is contributing to *isp‐1* longevity. To test this idea, it would be important to examine the effect of disrupting both axon regeneration pathway genes that are and are not required for *isp‐1* longevity on axon regeneration in *isp‐1* worms.

A limited number of previous studies have examined the effect of MLK‐1, MEK‐1 or KGB‐1 on lifespan and none of these genes appear in the GenAge database (https://genomics.senescence.info/genes/index.html), which chronicles genes that have been shown to affect lifespan. A previous study examining the mechanisms by which intermittent fasting extends lifespan found that fasting results in activation of KGB‐1 and that disruption of any components of the MLK‐1/MEK‐1/KGB‐1/FOS‐1 pathway decreases the magnitude of lifespan extension resulting from intermittent fasting (Uno et al. 2013). The same study demonstrated that KGB‐1 is also required for the upregulation of DAF‐16 target genes in response to fasting, indicating that KGB‐1 can activate both FOS‐1 and DAF‐16 to alter transcription (Uno et al. 2013). The MLK‐1/MEK‐1/KGB‐1 pathway has also been shown to be required for entry into dauer in response to environmental stressors (Dogra et al. 2022). Dauer is an alternative diapause developmental state in which worms are stress resistant and survive for multiple months.

MEK‐1 has been shown to be required for lifespan extension resulting from exposure to *P. freudenreichii* bacteria (Kwon et al. 2016). *P. freudenreichii* bacteria increases the expression of *mek‐1* and extends worm lifespan in wild‐type worms but not in *mek‐1* mutants. MEK‐1 has also been shown to be required for the compounds catapol (Seo et al. 2015) or curcumin (Liao et al. 2011) to increase lifespan.

KGB‐1 has been found to have different effects on stress resistance, lifespan and gene expression during development and during adulthood. While RNAi targeting *kgb‐1* decreases resistance to heavy metals during larval development, it enhances resistance during adulthood and increases lifespan (Twumasi‐Boateng et al. 2012). Treatment with *vhp‐1* RNAi during development activates KGB‐1 and causes nuclear localization of DAF‐16 and upregulation of the DAF‐16 target gene SOD‐3, in a KGB‐1‐dependent manner. Conversely, *vhp‐1* RNAi treatment during adulthood decreases DAF‐16 nuclear localization and SOD‐3 expression, also in a KGB‐1 dependent manner. Combined it was concluded that the effect of KGB‐1 on lifespan and infection resistance is dependent on DAF‐16 while its effect on heavy metal resistance is DAF‐16‐independent (Twumasi‐Boateng et al. 2012).

*kgb‐1* deletion mutants were found to have a decreased lifespan (but increased survival of osmotic stress) (Gerke et al. 2014). This may be due to the detrimental effect of *kgb‐1* disruption during development outweighing the positive effect during adulthood. *kgb‐1* deletion mutants were also found to have decreased survival following recovery from L1 diapause (Roux et al. 2016) and have a decreased ability to survive and develop to adulthood under ER stress (tunicamycin) or heavy metal stress (cadmium) (L. Liu et al. 2018).

## 4 Conclusion

In this work, we show that the kinase MAK‐2 is required for lifespan extension and enhanced resistance to stress in long‐lived *isp‐1* mutants. We further find that the long lifespan of *isp‐1* mutants is also dependent on a MLK‐1/MEK‐1/KGB‐1 kinase signaling pathway. Our results suggest that DAF‐16 acts independently of these two pathways to promote longevity in *isp‐1* worms (Figure S17). Overall, this work demonstrates the importance of kinase signaling in the lifespan extension that results from the mild impairment of mitochondrial function.

## 5 Methods

### 5.1 Strains

Strains were maintained on nematode growth media (NGM) plates seeded with OP50 bacteria at 20°C. The following strains were used in this study: N2—wild‐type, JVR650 *mak‐2(gk1110)*, JVR171 *isp‐1(qm150)*, JVR655 *isp‐1(qm150);mak‐2(gk1110)*, *ife‐2 (ok306)*, *clk‐1(qm30)*, *eat‐2(ad1116)*, *osm‐5(p813)*, *nuo‐6(qm200)*, *daf‐2(e1370)*, *glp‐1(e2141), TJ356 zIs356 [daf‐16p:daf‐16::GFP]*, and *isp‐1; zIs356 [daf‐16p:daf‐16::GFP]*. All strains were outcrossed to N2 a minimum of three times.

### 5.2 Fertility—Brood Size Assay

Brood size was determined by placing individual L4 worms onto NGM plates. Worms were transferred daily to new plates until progeny production ceased. The resulting progeny was allowed to develop to adulthood before quantification. Three biological replicates of five animals for each strain were completed.

### 5.3 Post‐Embryonic Development Time

Post‐embryonic development time (PED) was assessed by transferring newly hatched L1 worms to a new plate. Starting at 40 h after hatching, worms were scored approximately every 2 h and worms that reached young adulthood were removed from the plate. The hours from hatching to young adulthood were measured as the PED time. Three biological replicates of 20 animals each were completed.

### 5.4 Movement—Thrashing Rate

Young adult worms were transferred to OP50 *E. coli‐*seeded 60 mm NGM plates and a volume of 1 mL of M9 buffer was added to the worms. The worms were left to acclimatize for 1 min before capturing 1 min video at 14 FPS using WormLab (MBF Bioscience). The videos were analyzed using wrMTrck plugin for the open‐source image processing software, Fiji. Worm tracks were only considered for analysis when they included at least 420 frames (30 s). The results were pooled from three biological replicates.

### 5.5 Defecation Cycle Length

Defecation cycle length was determined by measuring the time between consecutive pBoc contractions in young adult worms with at least 10 worms per replicate on NGM plates fully covered with OP50 *E. coli* . To minimize the effects of ambient laboratory temperature, defecation was measured on water filled chambers that had been incubated at 20°C and the lids of the plates containing the worms were not removed. Three biological replicates were completed.

### 5.6 Osmotic Stress Assay

NGM plates containing 500 mM NaCl were poured and allowed to dry on the bench before storing at 4°C. One day before the experiment, 200 μL of 5X OP50 *E. coli* was seeded onto each plate and allowed to dry overnight. The next day, young adult worms were picked into the NGM plates containing 500 mM NaCl and incubated at 20°C for 48 h before scoring survival. Worms with internal hatching were censored and excluded from the total number of deaths. Three biological replicates were completed.

### 5.7 Heat Stress Assay

NGM plates were freshly seeded with 50 μL of 10X OP50 *E. coli* . Once dried, young adult worms were transferred to the plates and incubated at 37°C. Survival was monitored every 2 h for a total of 8 h. Three biological replicates were completed.

### 5.8 Chronic Oxidative Stress Assay

Resistance to chronic oxidative stress was assessed by exposing worms to 4 mM paraquat (Methyl viologen dichloride hydrate). Paraquat freshly dissolved in ddH_2_O was added to NGM before pouring and 100 μM 5‐fluoro‐2′‐deoxyuridine (FUdR) was included in the plates to prevent internal hatching that is caused by exposure to paraquat. The plates were allowed to dry overnight before storage at 4°C. One day before the experiment, the paraquat plates were seeded with 200 μL of 10X OP50 *E. coli* and allowed to dry overnight. The next day, young adult worms were transferred to these plates and survival was monitored every day until death. Three biological replicates were completed.

### 5.9 Lifespan

Lifespan assays were conducted at 20°C. To prevent progeny from developing to adulthood, lifespan plates included 25 μM 5‐fluoro‐2′‐deoxyuridine (FUdR) (Van Raamsdonk and Hekimi 2011). Experimenters were blinded to the genotype of strains being assayed. Worms were censored if they showed internal hatching, externalization of internal organs, or protruding vulva. Worms were checked every 2–3 days by gentle prodding using a platinum worm pick.

### 5.10 RNA Interference

RNA interference bacteria was grown in 2YT containing 50 μg/mL Carbenicillin for 16 h at 37°C on a shaker set at 250 RPM. The bacteria was concentrated 2X and immediately used to seed plates. RNAi plates were freshly made from 60 mm NGM plates containing 1 mM IPTG and 50 μg/mL Carbenicillin. Two sets of RNAi plates were made, ones without FUdR and ones containing 50 μM of FUdR. Plates were left to dry for 48 h before seeding, at which point each plate was seeded with 600 μM of 2X bacteria. Plates were left on benchtop until dry, and then stored at 4°C until needed.

### 5.11 RNAi Lifespan

For assessing the role of axon regeneration pathway genes on *isp‐1* lifespan, L4 worms were placed on RNAi plates with no FUdR. The next day all the worms were transferred to a fresh plate to allow egg laying for 24 h. After 24 h, all of the egg laying worms were removed. Once the eggs hatched and developed to the L4 stage, they were transferred on to the RNAi FUdR plates. Thereafter, worms were transferred only as needed. For assessing the contribution of MAK‐2 to the lifespan of long‐lived mutant strains, worms were grown until they were at the pre‐fertile young adult stage before initiating treatment with *mak‐2* RNAi. For both experiments, all worms were maintained and scored at 20°C, unless otherwise specified. Notably, for *glp‐1* and its corresponding wild‐type control, worms were allowed to develop at 25°C before being shifted to 20°C at adulthood. Worms were checked 3 times a week for survival. Survival was checked, based on movement, pharyngeal pumping and defecation. If the worm did not show any signs of movement, it was gently poked on the tail followed by the head to initiate movement. If the worm still did not move after this, it was marked as dead. Worms that crawled off the plate or died from expulsion of internal organs were censored from the dataset.

### 5.12 Quantitative RT‐PCR

Total RNA was extracted from synchronized *C. elegans* young adults using TRIzol Reagent (Thermo Fisher Scientific). Following DNase treatment of the RNAs, cDNA synthesis was performed using the High‐Capacity cDNA Reverse Transcription Kit (Applied Biosystems) according to the manufacturer's protocol. Quantitative PCR was conducted using SYBR Green Master Mix (Applied Biosystems) on a ViiA 7 Real‐Time PCR System (Applied Biosystems). Gene expression levels were normalized to an endogenous control *act‐3* and analyzed using the ΔΔCt method. Three biological replicates were completed.

### 5.13 RNA Sequencing

Synchronized pre‐fertile young adult *C. elegans* populations were harvested using M9 buffer and washed 3 times with ice‐cold M9 to remove bacteria. Worm pellets were resuspended in TRIzol Reagent (Thermo Fisher Scientific), flash‐frozen in liquid nitrogen and then stored at −80°C until RNA extraction. Three biological replicates at different time were collected. Once all samples for the three replicates had been collected, RNA isolation from all the samples was performed at the same time. Samples were subjected to three freeze–thaw cycles to enhance lysis. After thawing, samples were vortexed, incubated at room temperature, and mixed with chloroform. Following phase separation by centrifugation at 12,000 × *g* for 15 min at 4°C, the aqueous phase was transferred, and RNA was precipitated with isopropanol. Pellets were washed twice with 75% ethanol, followed by an additional wash with 100% ethanol. RNA was air‐dried and resuspended in RNase‐free water. RNA concentration and purity were assessed using a Nanodrop spectrophotometer and subsequently measured using an Agilent Bioanalyzer prior to library preparation. RNA sequencing was performed by Genome Quebec.

### 5.14 Bioinformatic Analysis

Bioinformatic analyses were performed by the McGill Genome Centre. Adaptor sequences and low‐quality score bases (Phred score < 30) were first trimmed using *Trimmomatic* (Bolger et al. 2014). The resulting reads were aligned to the WBcel235 assembly of the *C. elegans* reference genome, using *STAR* (Dobin et al. 2013). Read counts were obtained using *HTSeq* (Anders et al. 2015) with parameters *‐m intersection‐nonempty ‐stranded = reverse*. Lowly‐expressed genes with an average read count lower than 10 across all samples were excluded from all downstream analyses. ComBat‐seq was used to correct for batch effects (Zhang et al. 2020). *edgeR*'s TMM algorithm was used to normalize raw counts (Robinson and Oshlack 2010) which were then transformed to log2‐counts per million (log2CPM) using the *voom* function implemented in the *limma* R package (Ritchie et al. 2015). A linear model was fitted using the *lmfit* function to assess differences in gene expression levels. The Benjamini‐Hochberg method was used to correct nominal *p*‐values for multiple testing. Gene ontology analyses were performed using ShinyGO 0.81 (https://bioinformatics.sdstate.edu/go/) and GOrilla (https://cbl‐gorilla.cs.technion.ac.il/). Weighted Venn diagrams were generated using BioVenn (https://www.biovenn.nl/).

### 5.15 Transcription Factor Analysis

To identify potential upstream transcriptional regulators in *isp‐1* worms, we performed transcription factor (TF) activity inference and promoter motif enrichment analysis.

For TF activity inference, analyses were carried out in R (version 4.5.1) using the decoupleR package (version 2.16.0) (Badia et al. 2022), together with the CelEsT framework (v1.1). CelEsT is based on a curated *C. elegans* TF–target regulatory network that integrates multiple sources of evidence, including ChIP‐seq, DNA‐binding motifs, and eY1H data (Perez 2025). The input consisted of gene IDs and their corresponding *t*‐statistics, based on pre‐filtered differentially expressed gene sets (separately for upregulated and downregulated genes). Gene IDs were first mapped to the CelEsT annotation, and genes that could not be mapped or were not present in the regulatory network were excluded prior to analysis.

Two complementary linear modeling approaches were used: univariate linear modeling (ULM) and multivariate linear modeling (MLM). ULM was implemented in R using decoupleR::run_ulm() and evaluates each TF regulon independently (minimum regulon size = 5). MLM results were obtained from the default implementation in the CelEsT application, where multiple TF regulons are modeled simultaneously to estimate TF‐specific effects while accounting for overlap among target genes. Both methods were applied using the same underlying regulatory network.

To provide sequence‐based support, promoter motif enrichment analysis was performed on the differentially expressed gene sets. Promoter regions were defined based on the *C. elegans* reference genome (PRJNA13758, WormBase WS298) and corresponding annotation file, by extracting sequences upstream of the transcription start site (TSS). Both 500 bp and 1000 bp upstream windows were used.

Promoter sequences from the target gene sets were used as the foreground, while promoter sequences from all RNA‐seq detected genes excluding the corresponding foreground genes were used as the background.

Motif enrichment analysis was performed using AME from the MEME Suite (v5.5.7), using Fisher's exact test with average odds scoring (Bailey et al. 2015). The motif database used was the *C. elegans* subset from CIS‐BP (version 1.02) (Weirauch et al. 2014). All analyses were conducted on the rorqual4 cluster of the Digital Research Alliance of Canada.

To reduce potential technical bias, we compared GC content distributions between foreground and background sequences and did not observe substantial differences. In addition, promoter sequence redundancy was assessed and no significant duplication was detected.

Because operon structure in *C. elegans* can result in multiple genes sharing a single promoter, which may affect promoter definition, we annotated genes using operon information from the reference annotation (WormBase WS298). Based on gene position within operons (first gene vs. downstream gene), downstream genes were removed in a sensitivity analysis, and motif enrichment analysis was repeated. Results before and after filtering were compared to evaluate the impact of operon structure. This filtering step was applied only in the motif analysis.

### 5.16 Statistical Analysis

Statistical analyses were performed using GraphPad Prism version 9.0. A log‐rank test was used for lifespan, chronic oxidative stress, bacterial pathogen stress, and post‐embryonic development time assays. For all other assays, a two‐way ANOVA with Šidák's multiple comparisons test was used. Error bars indicate standard error of the mean.

## Author Contributions

Conceptualization: J.M.V.R. Methodology: U.A., A.A., S.Z., A.A.T.G., E.C., J.G., M.M.P., G.F.B., A.P., J.M.V.R. Investigation: U.A., A.A., S.Z., A.A.T.G., E.C., J.G., M.M.P., G.F.B., A.P., J.M.V.R. Analysis: U.A., A.A., S.Z., A.A.T.G., E.C., J.G., M.M.P., G.F.B., A.P., J.M.V.R. Validation: U.A., A.A., S.Z., A.A.T.G., E.C., J.G., M.M.P., G.F.B., A.P., J.M.V.R. Visualization: U.A., A.A., S.Z., A.A.T.G., E.C., J.G., M.M.P., G.F.B., A.P., J.M.V.R. Writing – original draft: J.M.V.R. Writing – review and editing: U.A., A.A., S.Z., A.A.T.G., E.C., J.G., M.M.P., G.F.B., A.P., J.M.V.R. Supervision: J.M.V.R.

## Funding

This work was supported by the Canadian Institutes of Health Research and Natural Sciences and Engineering Research Council of Canada.

## Conflicts of Interest

The authors declare no conflicts of interest.

## Supporting information


**Figure S1:** Role of *mak‐2* in the longevity of long‐lived mutants. To assess the contribution of MAK‐2 to the long lifespan of long‐lived genetic mutants, we treated worms with *mak‐2* RNAi from young adulthood and measured lifespan. Knocking down *mak‐2* significantly decreased the lifespan of *isp‐1* worms but did not reduce the longevity of any other long‐lived mutants, though a trend toward decreased lifespan was observed for *ife‐2* and *daf‐2*. Interestingly, *mak‐2* RNAi increased the lifespan of *osm‐5* and *glp‐1* mutants. Three biological replicates were performed. Statistical significance was assessed using a two‐way ANOVA with Šidák's multiple comparisons test or the log‐rank test. *p < 0.05, **p < 0.01. Raw lifespan data can be found in Table S1.
**Figure S2:** *mak‐2* is not required for lifespan extension by RNAi targeting mitochondrial electron transport chain genes. Wild‐type and *mak‐2* mutants were treated with RNAi targeting (A) *nuo‐2* (complex I), (B) *cyc‐1* (complex III) or (C) *cco‐1* (complex IV). In each case, RNAi knockdown of the gene encoding the mitochondrial electron transport chain protein increased lifespan in both wild‐type and *mak‐2* worms. The magnitude of lifespan extension was not diminished in *mak‐2* mutants compared to wild‐type worms. Statistical significance was assessed using the log‐rank test. ****p < 0.0001. Raw lifespan data can be found in Table S1.
**Figure S3:** Disruption of *mak‐2* does not increase ROS levels in *isp‐1* worms. *isp‐1* and *isp‐1;mak‐2* worms were stained with dihydroethidium (DHE). No differences in DHE staining were observed.
**Figure S4:** Activation of ATFS‐1 target genes in *isp‐1* mutants is not dependent on MAK‐2. (A) Heat map showing the expression of high confidence ATFS‐1 target genes in wild‐type, *mak‐2, isp‐1* and *isp‐1;mak‐2* worms. The upregulation of ATFS‐1 target genes in *isp‐1* worms is not affected by the disruption of *mak‐2*. (B) Examining the expression of the highest confidence ATFS‐1 target genes reveals that disruption of *mak‐2* does not decrease their expression in *isp‐1* worms or in a wild‐type background. Combined, this suggests that ATFS‐1 target genes are activated in *isp‐1* worms independently of MAK‐2. Statistical significance was assessed using a two‐way ANOVA with Šidák's multiple comparisons test in panel B. *p < 0.05, ****p < 0.0001.
**Figure S5:** Transcriptional changes in *isp‐1* and *isp‐1;mak‐2* mutants. RNA sequencing was used to examine gene expression changes in *isp‐1* and *isp‐1;mak‐2* mutants with wild‐type and *mak‐2* worms as controls. (A) The principal component analysis (PCA) plot demonstrates distinct clustering of the three biological replicates from each strain indicating clear differences in gene expression. (B) Differentially expressed genes in *isp‐1* worms compared to wild‐type worms. (C) Differentially expressed genes in *isp‐1;mak‐2* worms compared to wild‐type worms.
**Figure S6:** Transcriptional changes in *mak‐2* mutants. Gene expression changes in *mak‐2* mutants compared to wild‐type worms were determined using RNA sequencing. (A) Volcano plot comparing gene expression in *mak‐2* and wild‐type worms. (B) Heat map showing differentially expressed genes between *mak‐2* and wild‐type worms. Gene Ontology (GO) enrichment analysis for genes that are significantly upregulated (C) or downregulated (D) in *mak‐2* deletion mutants compared to wild‐type worms.
**Figure S7:** Enrichment analysis for genes upregulated in *isp‐1* worms in a *mak‐2*‐dependent manner. Enrichment analysis was performed using Gene Ontology enRIchment anaLysis and visuaLizAtion tool (GOrilla: https://cblgorilla.cs.technion.ac.il/). The genes analyzed were those found to be significantly upregulated in *isp‐1* worms but not *isp‐1;mak‐2* worms and not downregulated in *mak‐2* mutants.
**Figure S8:** Enrichment analysis for genes downregulated in *isp‐1* worms in a *mak‐2*‐dependent manner. Enrichment analysis was performed using Gene Ontology enRIchment anaLysis and visuaLizAtion tool (GOrilla: https://cbl‐gorilla.cs.technion.ac.il/). The genes analyzed were those found to be significantly downregulated in *isp‐1* worms but not *isp‐1;mak‐2* worms and not upregulated in *mak‐2* mutants.
**Figure S9:** No enrichment of ROS‐modulated genes among genes that are differentially expressed in *isp‐1* worms in a MAK‐2‐dependent manner. RNA sequencing results from this study were compared to a published microarray study examining genes expression in worms treated with a lifespan‐extending dose of 0.1 mM paraquat (Yee et al. 2014 *Cell*). (A) There was a significant degree of overlap between differentially expressed genes in *isp‐1* worms from this study and differentially expressed genes in *isp‐1* worms from Yee et al. (B) There was also a significant degree of overlap between differentially expressed genes in *isp‐1* worms from this study and genes differentially expressed after treatment with 0.1 mM paraquat from Yee et al. (C) In contrast, genes from this study that were differentially expressed in *isp‐1* worms in a MAK‐2‐dependent manner were underrepresented among genes differentially expressed after treatment with 0.1 mM paraquat from Yee et al. This suggests that the MAK‐2‐dependent differentially expressed genes in *isp‐1* worms are not mediated by elevated ROS.
**Figure S10:** Expression of axonal regeneration pathway components in *isp‐1* worms. The expression of axonal regeneration pathway component genes was measured by analyzing data from RNA sequencing. The levels of *mak‐2* were unaffected in *isp‐1* worms. *isp‐1* worms exhibited a small but significant increase in the levels of *dlk‐1, pmk‐3, cebp‐1, fos‐1* and *vhp‐1*. Statistical significance was determined using a two‐way ANOVA with Dunnett's multiple comparisons test. *p < 0.05, **p < 0.01, ****p < 0.0001.
**Figure S11:** Expression of genes that are upregulated in *isp‐1* worms in a MAK‐2‐dependent manner is not significantly decreased by disruption of genes in the MLK‐1/MEK‐1/KGB‐1/FOS‐1 pathway. The left column shows the expression of genes that are upregulated in *isp‐1* worms in a MAK‐2‐dependent manner in wild‐type, *mak‐2, isp‐1* and *isp‐1;mak‐2* worms. Data was obtained from the RNA‐seq results. In each case, the gene is upregulated in *isp‐1* worms and the expression is reduced by disruption of *mak‐2*. The left column exhibits the expression of these same genes in worms treated with RNAi targeting genes in the MLK‐1/MEK‐1/KGB‐1/FOS‐1 pathway. Although a trend toward decrease was observed in some cases, none of the RNAi treatments resulted in a significant decrease in expression. This suggests that MAK‐2 modulates the expression of these genes independent of the MLK‐1/MEK‐1/KGB‐1/FOS‐1 pathway. Statistical significance was assessed using a two‐way ANOVA with Šidák's multiple comparisons test in the left column. *p < 0.05, **p < 0.01, ***p < 0.001, ****p < 0.0001.
**Figure S12:** Upregulation of CEBP‐1 target genes in *isp‐1* mutants is not dependent on *mak‐2*. (A) Genes upregulated in *isp‐1* worms do not show a significant enrichment of CEBP‐1 target genes. (B) Genes that are upregulated in *isp‐1* worms in a *mak‐2‐*dependent manner do not show a significant enrichment of CEBP‐1 target genes. (C) The CEBP‐1 target genes that are upregulated in *isp‐1* worms are mostly also upregulated in *isp‐1;mak‐2* worms. This suggests that the upregulation of these CEBP‐1 target genes in *isp‐1* worms is not mediated by MAK‐2. (D) Examples of CEBP‐1 target genes that are upregulated in *isp‐1* worms showing a lack of dependence on *mak‐2*. Statistical significance was assessed using a two‐way ANOVA with Šidák's multiple comparisons test. ns = not significant, ****p < 0.0001.
**Figure S13:** Upregulation of DAF‐16 target genes in *isp‐1* mutants is not dependent on MLK‐1/MEK‐1/KGB‐1 signaling pathway. The expression of DAF‐16 target genes was measured using quantitative RT‐PCR after treating *isp‐1* worms with RNAi targeting *mlk‐1, mek‐1, kgb‐1* or *fos‐1. daf‐16* RNAi was included as a control. All of the DAF‐16 target genes exhibited a trend toward increase in *isp‐1* worms compared to wild‐type worms. While *daf‐16* RNAi appeared to decrease the expression levels of the DAF‐16 target genes, RNAi targeting components of the MLK‐1/MEK‐1/KGB‐1 signaling pathway had minimal effect on the expression of DAF‐16 target genes. *fos‐1* RNAi significantly increased the expression of *dod‐3* and *sod‐3*. Three biological replicates were performed. Statistical significance was assessed using a one‐way ANOVA with Dunnett's multiple comparisons test. All groups were compared to the *isp‐1*—EV group. ns = not significant. *p < 0.05, ***p < 0.001.
**Figure S14:** Upregulation of DAF‐16 target genes in *isp‐1* mutants is not dependent on *mak‐2*. The expression of the top 50 consensus DAF‐16 target genes from Tepper et al. *Cell* 2013 was examined in wild‐type, *mak‐2, isp‐1* and *isp‐1;mak‐2* worms. (A) The majority of the DAF‐16 target genes are significantly upregulated in *isp‐1* worms. These target genes are also significantly upregulated in *isp‐1;mak‐2* mutants. This indicates that *mak‐2* is not required for the upregulation of DAF‐16 target genes in *isp‐1* worms. (B) The upregulation of example DAF‐16 target genes *ftn‐1, sod‐3, mtl‐1* and *dod‐3* in *isp‐1* worms is not decreased by the disruption of *mak‐2*. Statistical significance was assessed using a two‐way ANOVA with Šidák's multiple comparisons test. ns = not significant, **p < 0.01.
**Figure S15:** Nuclear localization of DAF‐16 is not increased by disruption of genes in the MLK‐1/MEK‐1/KGB‐1 pathway. The nuclear localization of DAF‐16 was examined using *zIs356 [daf‐16p:daf‐16::GFP]* worms. In a wild‐type background, DAF‐16::GFP is completely cytoplasmic under normal conditions. When these worms were starved, DAF‐16::GFP was found to go to the nucleus. In *isp‐1;zIs356 [daf‐16p:daf‐16::GFP]* worms, DAF‐16::GFP is mostly cytoplasmic with some DAF‐16::GFP moving to the nucleus. Treating *isp‐1;zIs356 [daf‐16p:daf‐16::GFP]* worms with RNAi targeting *mak‐2, mlk‐1, mek‐1* or *kgb‐1* did not increase nuclear localization of DAF‐16::GFP. Note that since nuclear localization of DAF‐16::GFP is minimal in *isp‐1;zIs356 [daf‐16p:daf‐16::GFP]* worms, it was not possible to assess whether RNAi knockdown of these genes diminished nuclear localization of DAF‐16.
**Figure S16:** Disruption of DAF‐16 decreases lifespan similarly in *isp‐1* and *isp‐1;mak‐2* mutants. To examine how the MAK‐2 and DAF‐16 pathways interact to determine the lifespan of *isp‐1* worms, we knocked down *daf‐16* in *isp‐1* and *isp‐1;mak‐2* worms and measured lifespan. We included wild‐type and *mak‐2* worms as controls. In every case, we found that *daf‐16* RNAi markedly decreased lifespan. The lifespan of all four strains was similar when treated with *daf‐16* RNAi. There was no additive effect of disrupting *mak‐2* and *daf‐16*. Statistical significance for the survival plots was determined using the log‐rank test.
**Figure S17:** Model for contribution of different signaling pathways to *isp‐1* lifespan. MAK‐2, MLK‐1, MEK‐1, KGB‐1 and DAF‐16 are all required for the extended longevity of *isp‐1* worms. Neither the MAK‐2 pathway nor the MLK‐1/MEK‐1/KGB‐1 pathway are required for the activation of DAF‐16 target genes in *isp‐1* worms. *isp‐1* worms do not show an enrichment of CEBP‐1 target genes and those CEBP‐1 target genes that are upregulated in *isp‐1* worms are largely independent of MAK‐2. Our data suggests that MAK‐2, DAF‐16 and the MLK‐1/MEK‐1/KGB‐1 pathway are acting independently to promote longevity in *isp‐1* worms. Genes upregulated in *isp‐1* worms include innate immunity genes, stress response genes and DAF‐16 target genes.

**Table S1:** Raw lifespan data.

**Table S2:** Differentially expressed genes from RNA sequencing experiment.

**Table S3:** Transcription factor analysis for MAK‐2 dependent genes.

## Data Availability

Raw lifespan data is included in Table S1. Other raw data will be provided upon request. All materials used in this manuscript are available to be shared with the scientific community. Requests for data or materials should be addressed to Jeremy Van Raamsdonk (jeremy.vanraamsdonk@mcgill.ca).

## References

[acel70537-bib-0001] Anders, S. , P. T. Pyl , and W. Huber . 2015. “HTSeq–A Python Framework to Work With High‐Throughput Sequencing Data.” Bioinformatics 31, no. 2: 166–169. 10.1093/bioinformatics/btu638. 25260700 PMC4287950

[acel70537-bib-0002] Anglas, U. , A. A. Tamez Gonzalez , M. M. Promi , A. AlOkda , A. Pacis , and J. M. Van Raamsdonk . 2025. “Elevated Mitochondrial Superoxide Promotes Longevity Through a Mitochondria‐To‐Nucleus Kinase Signaling Pathway.” *bioRxiv* .10.1016/j.redox.2026.10438542721646

[acel70537-bib-0003] Badia, I. M. P. , J. Velez Santiago , J. Braunger , et al. 2022. “decoupleR: Ensemble of Computational Methods to Infer Biological Activities From Omics Data.” Bioinformatics Advances 2, no. 1: vbac016. 10.1093/bioadv/vbac016. 36699385 PMC9710656

[acel70537-bib-0004] Bailey, T. L. , J. Johnson , C. E. Grant , and W. S. Noble . 2015. “The MEME Suite.” Nucleic Acids Research 43, no. W1: W39–W49. 10.1093/nar/gkv416. 25953851 PMC4489269

[acel70537-bib-0005] Baker, S. T. , K. J. Opperman , E. D. Tulgren , S. M. Turgeon , W. Bienvenut , and B. Grill . 2014. “RPM‐1 Uses Both Ubiquitin Ligase and Phosphatase‐Based Mechanisms to Regulate DLK‐1 During Neuronal Development.” PLoS Genetics 10, no. 5: e1004297. 10.1371/journal.pgen.1004297. 24810406 PMC4014440

[acel70537-bib-0006] Baruah, A. , H. Chang , M. Hall , et al. 2014. “CEP‐1, the *Caenorhabditis elegans* p53 Homolog, Mediates Opposing Longevity Outcomes in Mitochondrial Electron Transport Chain Mutants.” PLoS Genetics 10, no. 2: e1004097. 10.1371/journal.pgen.1004097. 24586177 PMC3937132

[acel70537-bib-0007] Bolger, A. M. , M. Lohse , and B. Usadel . 2014. “Trimmomatic: A Flexible Trimmer for Illumina Sequence Data.” Bioinformatics 30, no. 15: 2114–2120. 10.1093/bioinformatics/btu170. 24695404 PMC4103590

[acel70537-bib-0008] Borror, M. B. , M. Girotti , A. Kar , et al. 2022. “Inhibition of ATR Reverses a Mitochondrial Respiratory Insufficiency.” Cells 11, no. 11: 1731. 10.3390/cells11111731. 35681427 PMC9179431

[acel70537-bib-0009] Campos, J. C. , Z. Wu , P. D. Rudich , et al. 2021. “Mild Mitochondrial Impairment Enhances Innate Immunity and Longevity Through ATFS‐1 and p38 Signaling.” EMBO Reports 22: e52964. 10.15252/embr.202152964. 34617666 PMC8647147

[acel70537-bib-0010] Copeland, J. M. , J. Cho , T. Lo Jr. , et al. 2009. “Extension of Drosophila Life Span by RNAi of the Mitochondrial Respiratory Chain.” Current Biology: CB 19: 1591–1598. 10.1016/j.cub.2009.08.016. 19747824

[acel70537-bib-0011] Curtis, R. , G. O'Connor , and P. S. DiStefano . 2006. “Aging Networks in *Caenorhabditis elegans* : AMP‐Activated Protein Kinase (Aak‐2) Links Multiple Aging and Metabolism Pathways.” Aging Cell 5, no. 2: 119–126. 10.1111/j.1474-9726.2006.00205.x. 16626391

[acel70537-bib-0012] Das, P. , A. Aballay , and J. Singh . 2024. “Calcineurin Inhibition Enhances *Caenorhabditis elegans* Lifespan by Defecation Defects‐Mediated Calorie Restriction and Nuclear Hormone Signaling.” eLife 12: RP89572. 10.7554/eLife.89572. 39485281 PMC11530235

[acel70537-bib-0013] Dell'agnello, C. , S. Leo , A. Agostino , et al. 2007. “Increased Longevity and Refractoriness to ca(2+)‐Dependent Neurodegeneration in Surf1 Knockout Mice.” Human Molecular Genetics 16, no. 4: 431–444. 10.1093/hmg/ddl477. 17210671

[acel70537-bib-0014] Dillin, A. , A. L. Hsu , N. Arantes‐Oliveira , et al. 2002. “Rates of Behavior and Aging Specified by Mitochondrial Function During Development.” Science 298, no. 5602: 2398–2401. 10.1126/science.1077780. 12471266

[acel70537-bib-0015] Dobin, A. , C. A. Davis , F. Schlesinger , et al. 2013. “STAR: Ultrafast Universal RNA‐seq Aligner.” Bioinformatics 29, no. 1: 15–21. 10.1093/bioinformatics/bts635. 23104886 PMC3530905

[acel70537-bib-0016] Dogra, D. , W. Kulalert , F. C. Schroeder , and D. H. Kim . 2022. “Neuronal KGB‐1 JNK MAPK Signaling Regulates the Dauer Developmental Decision in Response to Environmental Stress in *Caenorhabditis elegans* .” Genetics 220, no. 1: iyab186. 10.1093/genetics/iyab186. 34726729 PMC8733477

[acel70537-bib-0017] Dues, D. J. , C. E. Schaar , B. K. Johnson , et al. 2017. “Uncoupling of Oxidative Stress Resistance and Lifespan in Long‐Lived Isp‐1 Mitochondrial Mutants in *Caenorhabditis elegans* .” Free Radical Biology & Medicine 108: 362–373. 10.1016/j.freeradbiomed.2017.04.004. 28392283 PMC5493208

[acel70537-bib-0018] Durieux, J. , S. Wolff , and A. Dillin . 2011. “The Cell‐Non‐Autonomous Nature of Electron Transport Chain‐Mediated Longevity.” Cell 144, no. 1: 79–91. 10.1016/j.cell.2010.12.016. 21215371 PMC3062502

[acel70537-bib-0019] El Bejjani, R. , and M. Hammarlund . 2012. “Neural Regeneration in *Caenorhabditis elegans* .” Annual Review of Genetics 46: 499–513. 10.1146/annurev-genet-110711-155550. PMC3700416 22974301

[acel70537-bib-0020] Feng, J. , F. Bussiere , and S. Hekimi . 2001. “Mitochondrial Electron Transport Is a Key Determinant of Life Span in *Caenorhabditis elegans* .” Developmental Cell 1, no. 5: 633–644. 11709184 10.1016/s1534-5807(01)00071-5

[acel70537-bib-0021] Gerke, P. , A. Keshet , A. Mertenskotter , and R. J. Paul . 2014. “The JNK‐Like MAPK KGB‐1 of *Caenorhabditis elegans* Promotes Reproduction, Lifespan, and Gene Expressions for Protein Biosynthesis and Germline Homeostasis but Interferes With Hyperosmotic Stress Tolerance.” Cellular Physiology and Biochemistry 34: 1951–1973. 10.1159/000366392. 25500773

[acel70537-bib-0022] Harris‐Gauthier, N. , A. Traa , A. AlOkda , et al. 2022. “Mitochondrial Thioredoxin System Is Required for Enhanced Stress Resistance and Extended Longevity in Long‐Lived Mitochondrial Mutants.” Redox Biology 53: 102335. 10.1016/j.redox.2022.102335. 35598379 PMC9126954

[acel70537-bib-0023] Hisamoto, N. , and K. Matsumoto . 2017. “Signal Transduction Cascades in Axon Regeneration: Insights From *C. elegans* .” Current Opinion in Genetics & Development 44: 54–60. 10.1016/j.gde.2017.01.010. 28213159

[acel70537-bib-0024] Hwang, A. B. , E. A. Ryu , M. Artan , et al. 2014. “Feedback Regulation via AMPK and HIF‐1 Mediates ROS‐Dependent Longevity in *Caenorhabditis elegans* .” Proceedings of the National Academy of Sciences of the United States of America 111, no. 42: E4458–E4467. 10.1073/pnas.1411199111. 25288734 PMC4210294

[acel70537-bib-0025] Jung, Y. , M. Artan , N. Kim , et al. 2021. “MON‐2, a Golgi Protein, Mediates Autophagy‐Dependent Longevity in *Caenorhabditis elegans* .” Science Advances 7, no. 49: eabj8156. 10.1126/sciadv.abj8156. 34860542 PMC8641931

[acel70537-bib-0026] Khan, M. H. , M. Ligon , L. R. Hussey , et al. 2013. “TAF‐4 Is Required for the Life Extension of Isp‐1, Clk‐1 and Tpk‐1 Mit Mutants.” Aging 5: 741–758. 24107417 10.18632/aging.100604 PMC3838777

[acel70537-bib-0027] Kim, K. W. , N. Thakur , C. A. Piggott , et al. 2016. “Coordinated Inhibition of C/EBP by Tribbles in Multiple Tissues Is Essential for *Caenorhabditis elegans* Development.” BMC Biology 14, no. 1: 104. 10.1186/s12915-016-0320-z. 27927209 PMC5141650

[acel70537-bib-0028] Ko, B. , and J. M. Van Raamsdonk . 2023. “RNA Sequencing of Pooled Samples Effectively Identifies Differentially Expressed Genes.” Biology‐Basel 12, no. 6: 812. 10.3390/biology12060812. 37372097 PMC10295764

[acel70537-bib-0029] Kwon, G. , J. Lee , and Y. H. Lim . 2016. “Dairy Propionibacterium Extends the Mean Lifespan of *Caenorhabditis elegans* via Activation of the Innate Immune System.” Scientific Reports 6: 31713. 10.1038/srep31713. 27531646 PMC4987649

[acel70537-bib-0030] Lakowski, B. , and S. Hekimi . 1996. “Determination of Life‐Span in *Caenorhabditis elegans* by Four Clock Genes.” Science 272, no. 5264: 1010–1013. 8638122 10.1126/science.272.5264.1010

[acel70537-bib-0031] Lee, S. J. , A. B. Hwang , and C. Kenyon . 2010. “Inhibition of Respiration Extends *C. elegans* Life Span via Reactive Oxygen Species That Increase HIF‐1 Activity.” Current Biology: CB 20, no. 23: 2131–2136. 10.1016/j.cub.2010.10.057. 21093262 PMC3058811

[acel70537-bib-0032] Lee, S. S. , R. Y. Lee , A. G. Fraser , R. S. Kamath , J. Ahringer , and G. Ruvkun . 2003. “A Systematic RNAi Screen Identifies a Critical Role for Mitochondria in *C. elegans* Longevity.” Nature Genetics 33, no. 1: 40–48. 10.1038/ng1056. 12447374

[acel70537-bib-0033] Liao, V. H. , C. W. Yu , Y. J. Chu , W. H. Li , Y. C. Hsieh , and T. T. Wang . 2011. “Curcumin‐Mediated Lifespan Extension in *Caenorhabditis elegans* .” Mechanisms of Ageing and Development 132, no. 10: 480–487. 10.1016/j.mad.2011.07.008. 21855561

[acel70537-bib-0034] Liu, L. , C. Ruediger , and M. Shapira . 2018. “Integration of Stress Signaling in *Caenorhabditis elegans* Through Cell‐Nonautonomous Contributions of the JNK Homolog KGB‐1.” Genetics 210, no. 4: 1317–1328. 10.1534/genetics.118.301446. 30291110 PMC6283176

[acel70537-bib-0035] Liu, X. , N. Jiang , B. Hughes , E. Bigras , E. Shoubridge , and S. Hekimi . 2005. “Evolutionary Conservation of the Clk‐1‐Dependent Mechanism of Longevity: Loss of mclk1 Increases Cellular Fitness and Lifespan in Mice.” Genes & Development 19, no. 20: 2424–2434. 10.1101/gad.1352905. 16195414 PMC1257397

[acel70537-bib-0036] Munkacsy, E. , M. H. Khan , R. K. Lane , et al. 2016. “DLK‐1, SEK‐3 and PMK‐3 Are Required for the Life Extension Induced by Mitochondrial Bioenergetic Disruption in *C. elegans* .” PLoS Genetics 12, no. 7: e1006133. 10.1371/journal.pgen.1006133. 27420916 PMC4946786

[acel70537-bib-0037] Nakata, K. , B. Abrams , B. Grill , et al. 2005. “Regulation of a DLK‐1 and p38 MAP Kinase Pathway by the Ubiquitin Ligase RPM‐1 Is Required for Presynaptic Development.” Cell 120, no. 3: 407–420. 10.1016/j.cell.2004.12.017. 15707898

[acel70537-bib-0038] Nix, P. , N. Hisamoto , K. Matsumoto , and M. Bastiani . 2011. “Axon Regeneration Requires Coordinate Activation of p38 and JNK MAPK Pathways.” Proceedings of the National Academy of Sciences of the United States of America 108, no. 26: 10738–10743. 10.1073/pnas.1104830108. 21670305 PMC3127873

[acel70537-bib-0039] Pastuhov, S. I. , N. Hisamoto , and K. Matsumoto . 2015. “MAP Kinase Cascades Regulating Axon Regeneration in *C. elegans* .” Proceedings of the Japan Academy. Series B, Physical and Biological Sciences 91, no. 3: 63–75. 10.2183/pjab.91.63. 25792136 PMC4410086

[acel70537-bib-0040] Perez, M. F. 2025. “CelEst: A Unified Gene Regulatory Network for Estimating Transcription Factor Activities in *C. elegans* .” Genetics 229, no. 3: iyae189. 10.1093/genetics/iyae189. 39705007 PMC11912867

[acel70537-bib-0041] Ramachandran, P. V. , M. Savini , A. K. Folick , et al. 2019. “Lysosomal Signaling Promotes Longevity by Adjusting Mitochondrial Activity.” Developmental Cell 48, no. 5: 685–696.e685. 10.1016/j.devcel.2018.12.022. 30713071 PMC6613828

[acel70537-bib-0042] Rea, S. L. , N. Ventura , and T. E. Johnson . 2007. “Relationship Between Mitochondrial Electron Transport Chain Dysfunction, Development, and Life Extension in *Caenorhabditis elegans* .” PLoS Biology 5, no. 10: e259. 10.1371/journal.pbio.0050259. 17914900 PMC1994989

[acel70537-bib-0043] Ritchie, M. E. , B. Phipson , D. Wu , et al. 2015. “Limma Powers Differential Expression Analyses for RNA‐Sequencing and Microarray Studies.” Nucleic Acids Research 43, no. 7: e47. 10.1093/nar/gkv007. 25605792 PMC4402510

[acel70537-bib-0044] Robinson, M. D. , and A. Oshlack . 2010. “A Scaling Normalization Method for Differential Expression Analysis of RNA‐Seq Data.” Genome Biology 11, no. 3: R25. 10.1186/gb-2010-11-3-r25. 20196867 PMC2864565

[acel70537-bib-0045] Roux, A. E. , K. Langhans , W. Huynh , and C. Kenyon . 2016. “Reversible Age‐Related Phenotypes Induced During Larval Quiescence in *C. elegans* .” Cell Metabolism 23, no. 6: 1113–1126. 10.1016/j.cmet.2016.05.024. 27304510 PMC5794336

[acel70537-bib-0046] Senchuk, M. M. , D. J. Dues , C. E. Schaar , et al. 2018. “Activation of DAF‐16/FOXO by Reactive Oxygen Species Contributes to Longevity in Long‐Lived Mitochondrial Mutants in *Caenorhabditis elegans* .” PLoS Genetics 14, no. 3: e1007268. 10.1371/journal.pgen.1007268. 29522556 PMC5862515

[acel70537-bib-0047] Seo, H. W. , S. M. Cheon , M. H. Lee , H. J. Kim , H. Jeon , and D. S. Cha . 2015. “Catalpol Modulates Lifespan via DAF‐16/FOXO and SKN‐1/Nrf2 Activation in *Caenorhabditis elegans* .” Evidence‐based Complementary and Alternative Medicine 2015: 524878. 10.1155/2015/524878. 25821490 PMC4363898

[acel70537-bib-0048] Soo, S. K. , and J. M. Van Raamsdonk . 2021. “High Confidence ATFS‐1 Target Genes for Quantifying Activation of the Mitochondrial Unfolded Protein Response.” Micropublication Biology 2021. 10.17912/micropub.biology.000484. PMC8527333 34693215

[acel70537-bib-0049] Tedeschi, A. , and F. Bradke . 2013. “The DLK Signalling Pathway–A Double‐Edged Sword in Neural Development and Regeneration.” EMBO Reports 14, no. 7: 605–614. 10.1038/embor.2013.64. 23681442 PMC3701236

[acel70537-bib-0050] Tulgren, E. D. , S. T. Baker , L. Rapp , A. M. Gurney , and B. Grill . 2011. “PPM‐1, a PP2Calpha/Beta Phosphatase, Regulates Axon Termination and Synapse Formation in *Caenorhabditis elegans* .” Genetics 189, no. 4: 1297–1307. 10.1534/genetics.111.134791. 21968191 PMC3241410

[acel70537-bib-0051] Twumasi‐Boateng, K. , T. W. Wang , L. Tsai , et al. 2012. “An Age‐Dependent Reversal in the Protective Capacities of JNK Signaling Shortens *Caenorhabditis elegans* Lifespan.” Aging Cell 11, no. 4: 659–667. 10.1111/j.1474-9726.2012.00829.x. 22554143 PMC3440580

[acel70537-bib-0052] Uno, M. , S. Honjoh , M. Matsuda , et al. 2013. “A Fasting‐Responsive Signaling Pathway That Extends Life Span in *C. elegans* .” Cell Reports 3, no. 1: 79–91. 10.1016/j.celrep.2012.12.018. 23352664

[acel70537-bib-0053] Van Raamsdonk, J. M. , and S. Hekimi . 2009. “Deletion of the Mitochondrial Superoxide Dismutase Sod‐2 Extends Lifespan in *Caenorhabditis elegans* .” PLoS Genetics 5, no. 2: e1000361. 10.1371/journal.pgen.1000361. 19197346 PMC2628729

[acel70537-bib-0054] Van Raamsdonk, J. M. , and S. Hekimi . 2011. “FUdR Causes a Twofold Increase in the Lifespan of the Mitochondrial Mutant Gas‐1.” Mechanisms of Ageing and Development 132, no. 10: 519–521. 10.1016/j.mad.2011.08.006. 21893079 PMC4074524

[acel70537-bib-0055] Van Raamsdonk, J. M. , and S. Hekimi . 2012. “Superoxide Dismutase is Dispensable for Normal Animal Lifespan.” Proceedings of the National Academy of Sciences of the United States of America 109, no. 15: 5785–5790. 10.1073/pnas.1116158109. 22451939 PMC3326508

[acel70537-bib-0056] Walter, L. , A. Baruah , H. W. Chang , H. M. Pace , and S. S. Lee . 2011. “The Homeobox Protein CEH‐23 Mediates Prolonged Longevity in Response to Impaired Mitochondrial Electron Transport Chain in *C. elegans* .” PLoS Biology 9, no. 6: e1001084. 10.1371/journal.pbio.1001084. 21713031 PMC3119657

[acel70537-bib-0057] Weirauch, M. T. , A. Yang , M. Albu , et al. 2014. “Determination and Inference of Eukaryotic Transcription Factor Sequence Specificity.” Cell 158, no. 6: 1431–1443. 10.1016/j.cell.2014.08.009. 25215497 PMC4163041

[acel70537-bib-0058] Wong, A. , P. Boutis , and S. Hekimi . 1995. “Mutations in the Clk‐1 Gene of *Caenorhabditis elegans* Affect Developmental and Behavioral Timing.” Genetics 139, no. 3: 1247–1259. 7768437 10.1093/genetics/139.3.1247 PMC1206454

[acel70537-bib-0059] Wu, Z. , M. M. Senchuk , D. J. Dues , et al. 2018. “Mitochondrial Unfolded Protein Response Transcription Factor ATFS‐1 Promotes Longevity in a Long‐Lived Mitochondrial Mutant Through Activation of Stress Response Pathways.” BMC Biology 16, no. 1: 147. 10.1186/s12915-018-0615-3. 30563508 PMC6298126

[acel70537-bib-0060] Yang, W. , and S. Hekimi . 2010a. “A Mitochondrial Superoxide Signal Triggers Increased Longevity in *Caenorhabditis elegans* .” PLoS Biology 8, no. 12: e1000556. 10.1371/journal.pbio.1000556. 21151885 PMC2998438

[acel70537-bib-0061] Yang, W. , and S. Hekimi . 2010b. “Two Modes of Mitochondrial Dysfunction Lead Independently to Lifespan Extension in *Caenorhabditis elegans* .” Aging Cell 9, no. 3: 433–447. 10.1111/j.1474-9726.2010.00571.x. 20346072

[acel70537-bib-0062] Yee, C. , W. Yang , and S. Hekimi . 2014. “The Intrinsic Apoptosis Pathway Mediates the Pro‐Longevity Response to Mitochondrial ROS in *C. elegans* .” Cell 157, no. 4: 897–909. 10.1016/j.cell.2014.02.055. 24813612 PMC4454526

[acel70537-bib-0063] Zhang, Y. , G. Parmigiani , and W. E. Johnson . 2020. “ComBat‐Seq: Batch Effect Adjustment for RNA‐Seq Count Data.” NAR Genomics and Bioinformatics 2, no. 3: lqaa078. 10.1093/nargab/lqaa078. 33015620 PMC7518324

