# Supplementary material for: MAK‐2 Kinase Is Required for Extended Longevity and Enhanced Stress Resistance Resulting From Mild Impairment of Mitochondrial Function in isp‐1 Mutants
Source: Aging Cell. 2026 May 13;25(5):e70537. doi: 10.1111/acel.70537 (PMC13169497; doi:10.1111/acel.70537)
Supplement: Supplementary file 1 — Figure S1: Role of mak‐2 in the longevity of long‐lived mutants. To assess the contribution of MAK‐2 to the long lifespan of long‐lived genetic mutants, we treated worms with mak‐2 RNAi from young adulthood and measured lifespan. Knocking down mak‐2 significantly decreased the lifespan of isp‐1 worms but did not reduce the longevity of any other long‐lived mutants, though a trend toward decreased lifespan was observed for ife‐2 and daf‐2. Interestingly, mak‐2 RNAi increased the lifespan of osm‐5 and glp‐1 mutants. Three biological replicates were performed. Statistical significance was assessed using a two‐way ANOVA with Šidák's multiple comparisons test or the log‐rank test. *p < 0.05, **p < 0.01. Raw lifespan data can be found in Table S1. Figure S2: mak‐2 is not required for lifespan extension by RNAi targeting mitochondrial electron transport chain genes. Wild‐type and mak‐2 mutants were treated with RNAi targeting (A) nuo‐2 (complex I), (B) cyc‐1 (complex III) or (C) cco‐1 (complex IV). In each case, RNAi knockdown of the gene encoding the mitochondrial electron transport chain protein increased lifespan in both wild‐type and mak‐2 worms. The magnitude of lifespan extension was not diminished in mak‐2 mutants compared to wild‐type worms. Statistical significance was assessed using the log‐rank test. ****p < 0.0001. Raw lifespan data can be found in Table S1. Figure S3: Disruption of mak‐2 does not increase ROS levels in isp‐1 worms. isp‐1 and isp‐1;mak‐2 worms were stained with dihydroethidium (DHE). No differences in DHE staining were observed. Figure S4: Activation of ATFS‐1 target genes in isp‐1 mutants is not dependent on MAK‐2. (A) Heat map showing the expression of high confidence ATFS‐1 target genes in wild‐type, mak‐2, isp‐1 and isp‐1;mak‐2 worms. The upregulation of ATFS‐1 target genes in isp‐1 worms is not affected by the disruption of mak‐2. (B) Examining the expression of the highest confidence ATFS‐1 target genes reveals that disruption of mak‐2 do [file ACEL-25-e70537-s003.pdf]

Supplementary Figures for

MAK-2 kinase is required for extended longevity and enhanced stress resistance resulting from mild impairment of mitochondrial function in *isp-1* mutants

Ulrich Anglas*, Abdelrahman AlOkda*, Shusen Zhu*, Aura A. Tamez González, Ekin Celtikcioglu, Jiaxi Guan, Maisha M. Promi, Grant F. Booth, Alain Pacis, Jeremy M. Van Raamsdonk

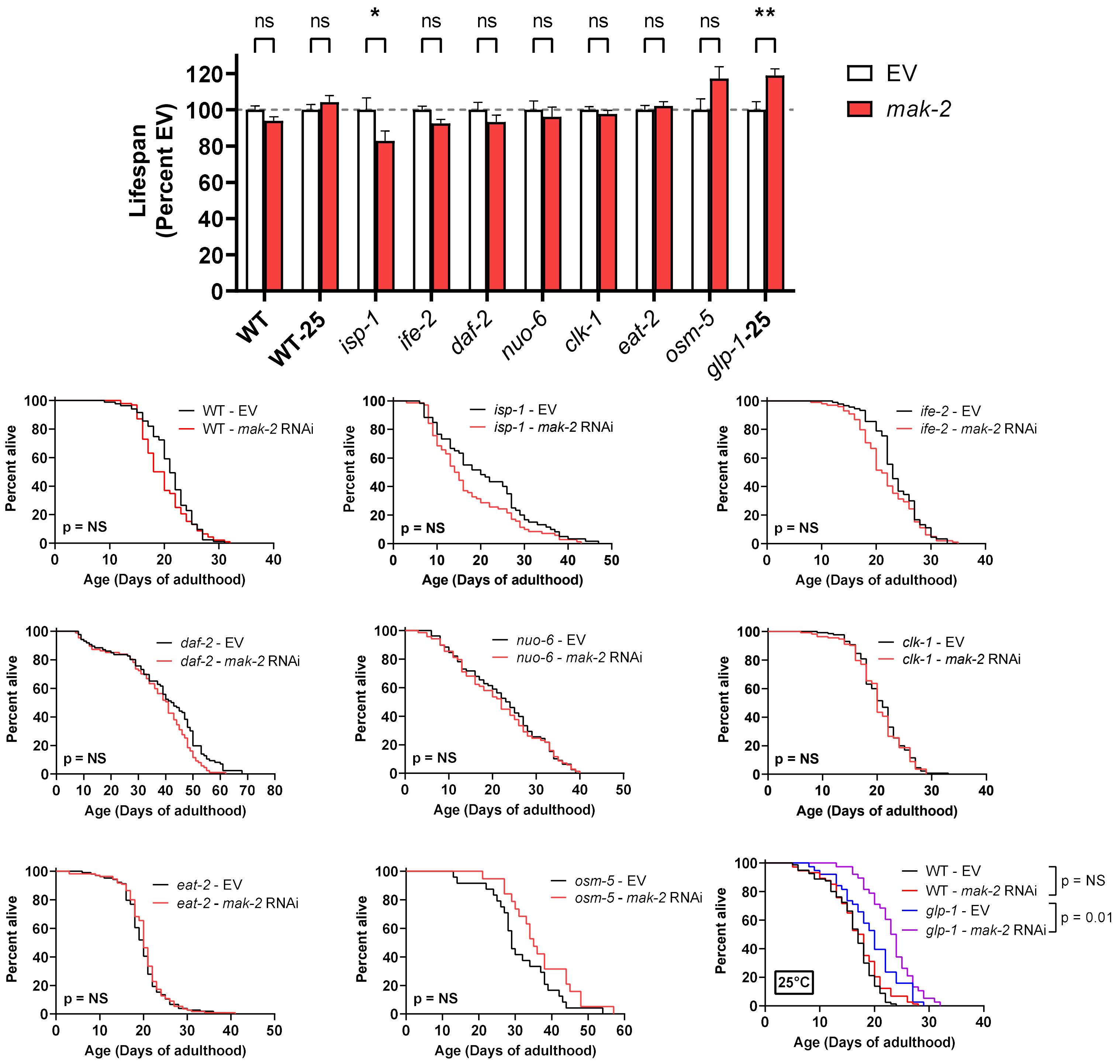

Figure S1. Role of *mak-2* in the longevity of long-lived mutants. To assess the contribution of MAK-2 to the long lifespan of long-lived genetic mutants, we treated worms with *mak-2* RNAi from young adulthood and measured lifespan. Knocking down *mak-2* significantly decreased the lifespan of *isp-1* worms but did not reduce the longevity of any other long-lived mutants, though a trend towards decreased lifespan was observed for *ife-2* and *daf-2*. Interestingly, *mak-2* RNAi increased the lifespan of *osm-5* and *glp-1* mutants. Three biological replicates were performed. Statistical significance was assessed using a two-way ANOVA with Šidák's multiple comparisons test or the log-rank test. *p<0.05, **p<0.01. Raw lifespan data can be found in **Table S1**.

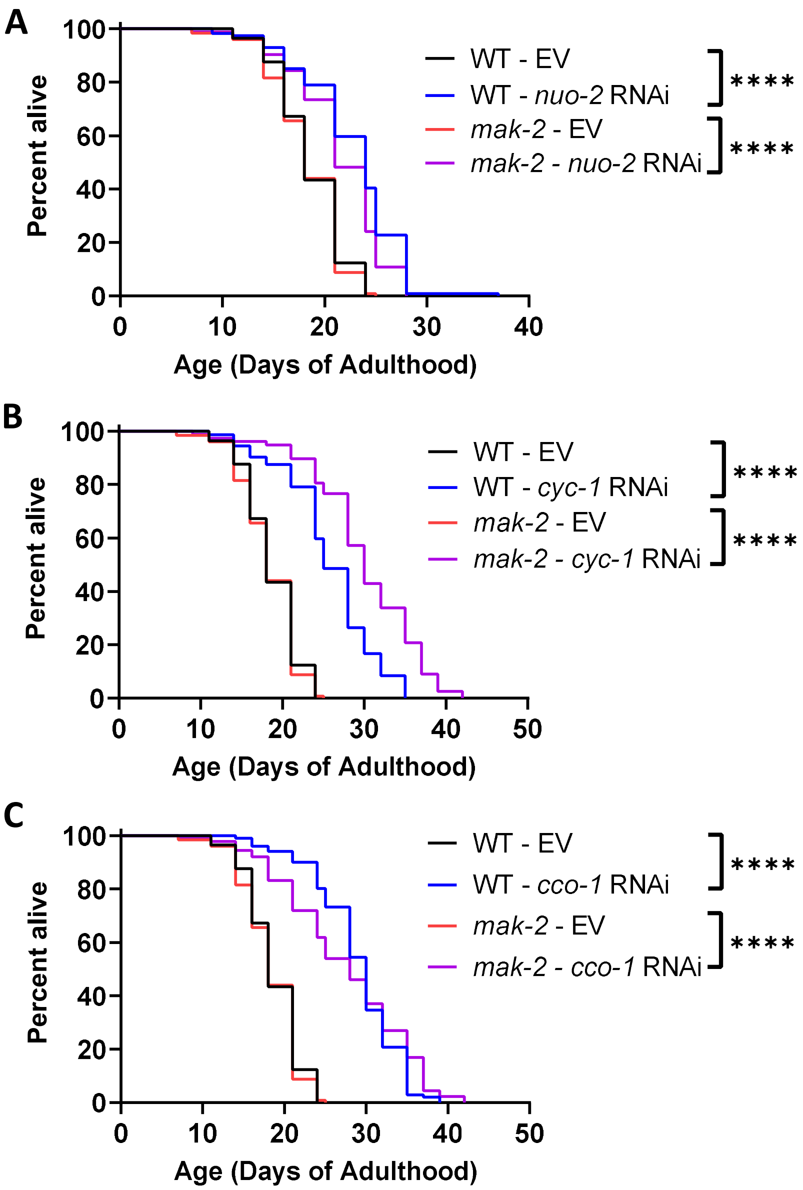

Figure S2. *mak-2* is not required for lifespan extension by RNAi targeting mitochondrial electron transport chain genes. Wild-type and *mak-2* mutants were treated with RNAi targeting (**A**) *nuo-2* (complex I), (**B**) *cyc-1* (complex III) or (**C**) *cco-1* (complex IV). In each case, RNAi knockdown of the gene encoding the mitochondrial electron transport chain protein increased lifespan in both wild-type and *mak-2* worms. The magnitude of lifespan extension was not diminished in *mak-2* mutants compared to wild-type worms. Statistical significance was assessed using the log-rank test. ****$p<0.0001$. Raw lifespan data can be found in **Table S1**.

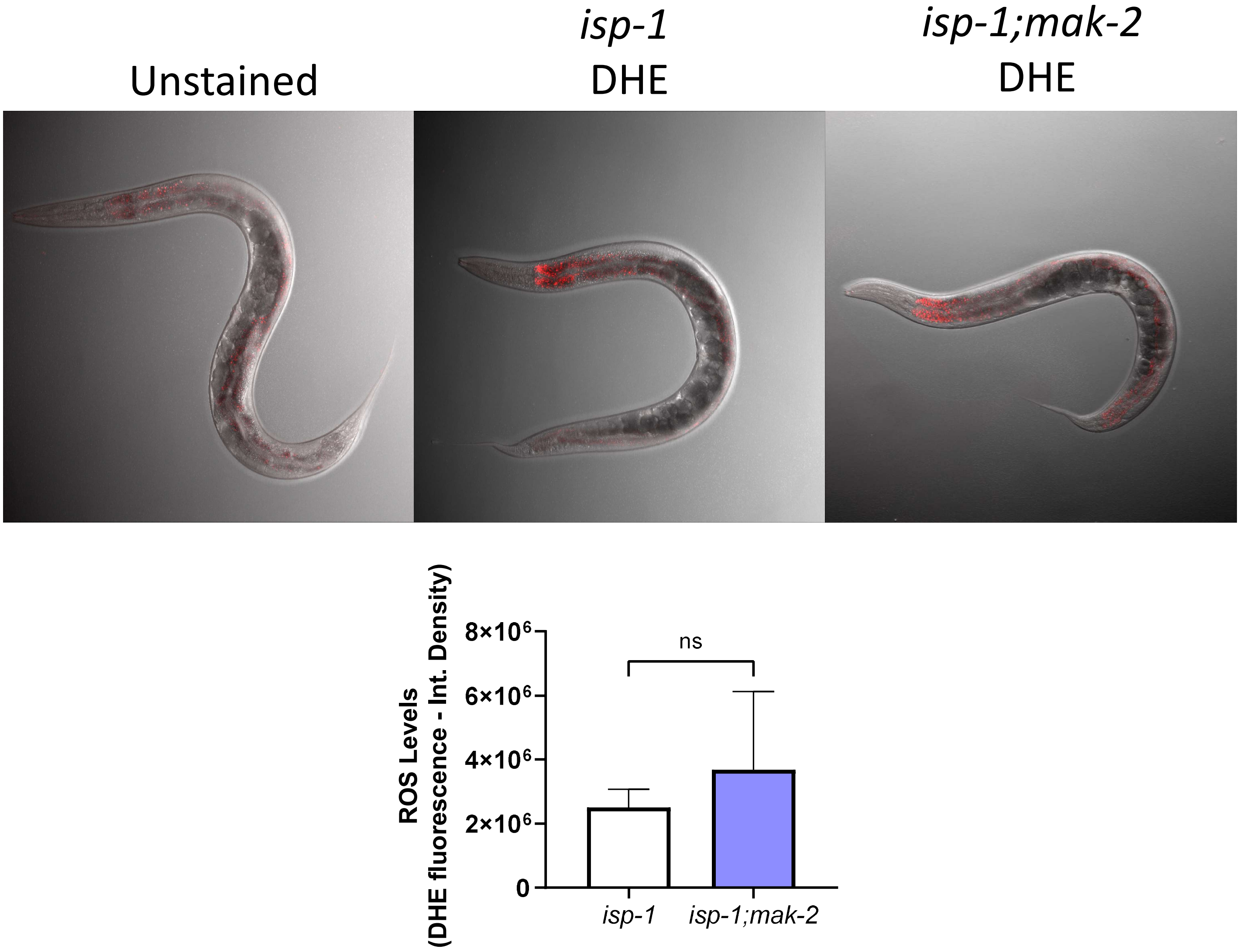

Figure S3 . Disruption of *mak-2* does not increase ROS levels in *isp-1* worms. *isp-1* and *isp-1;mak-2* worms were stained with dihydroethidium (DHE). No differences in DHE staining were observed.

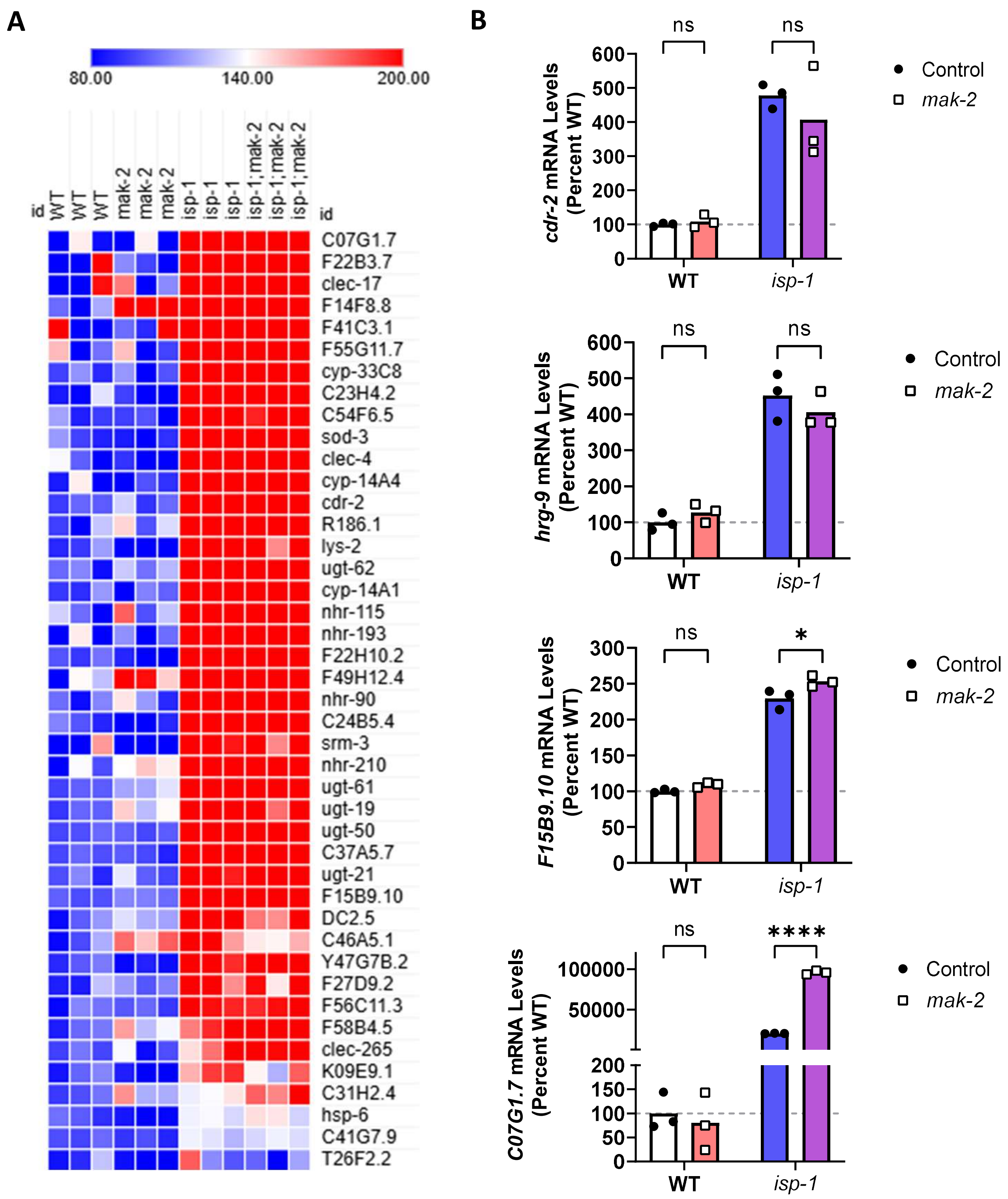

Figure S4. Activation of ATFS-1 target genes in *isp-1* mutants is not dependent on MAK-2. (**A**) Heat map showing the expression of high confidence ATFS-1 target genes in wild-type, *mak-2, isp-1* and *isp-1;mak-2* worms. The upregulation of ATFS-1 target genes in *isp-1* worms is not affected by the disruption of *mak-2.* (**B**) Examining the expression of the highest confidence ATFS-1 target genes reveals that disruption of *mak-2* does not decrease their expression in *isp-1* worms or in a wild-type background. Combined, this suggests that ATFS-1 target genes are activated in *isp-1* worms independently of MAK-2. Statistical significance was assessed using a two-way ANOVA with Šidák's multiple comparisons test in panel B. *p<0.05, ****p<0.0001.

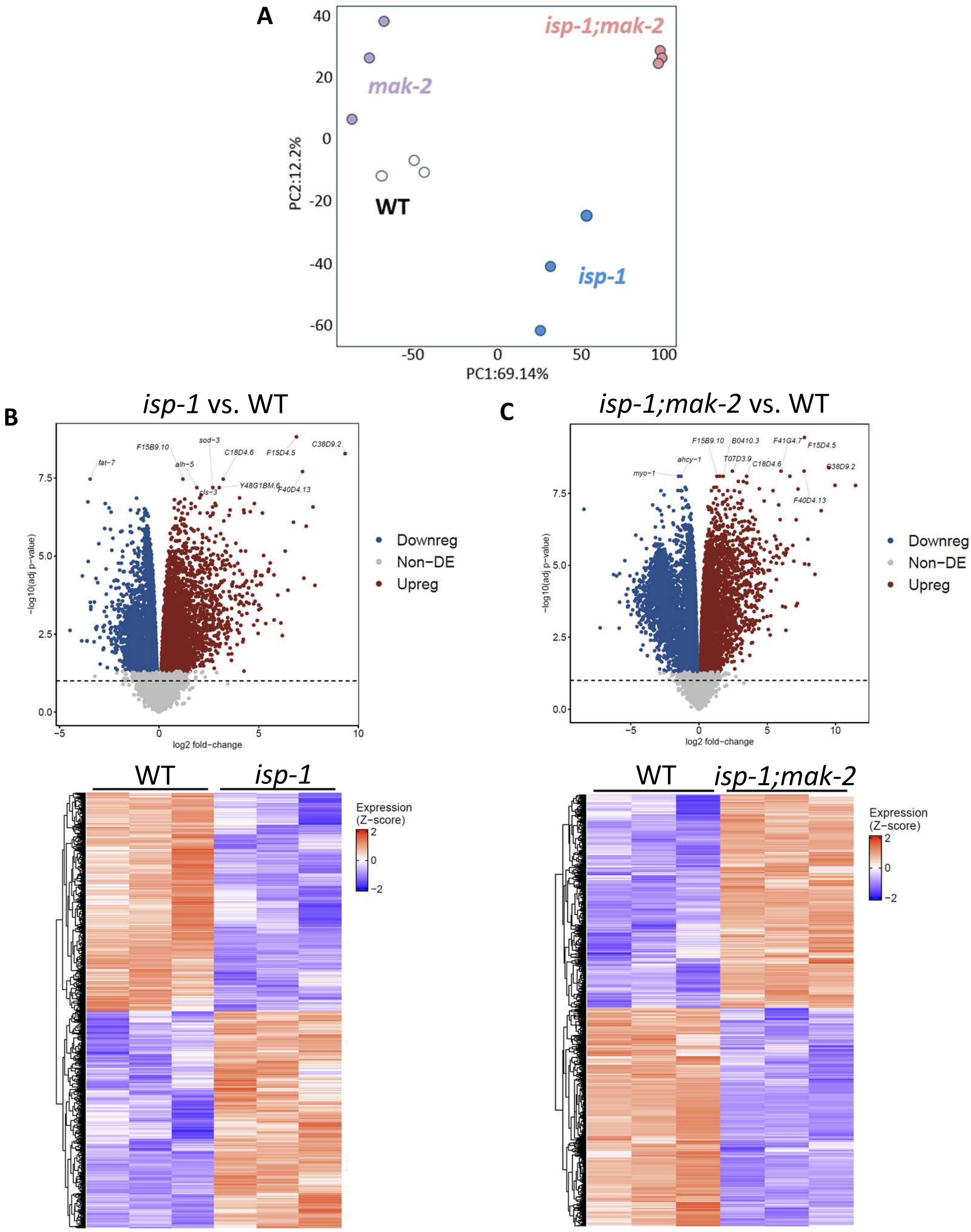

Figure S5. Transcriptional changes in *isp-1* and *isp-1;mak-2* mutants. RNA sequencing was used to examine gene expression changes in *isp-1* and *isp-1;mak-2* mutants with wild-type and *mak-2* worms as controls. (**A**) The principal component analysis (PCA) plot demonstrates distinct clustering of the three biological replicates from each strain indicating clear differences in gene expression. (**B**) Differentially expressed genes in *isp-1* worms compared to wild-type worms. (**C**) Differentially expressed genes in *isp-1;mak-2* worms compared to wild-type worms.

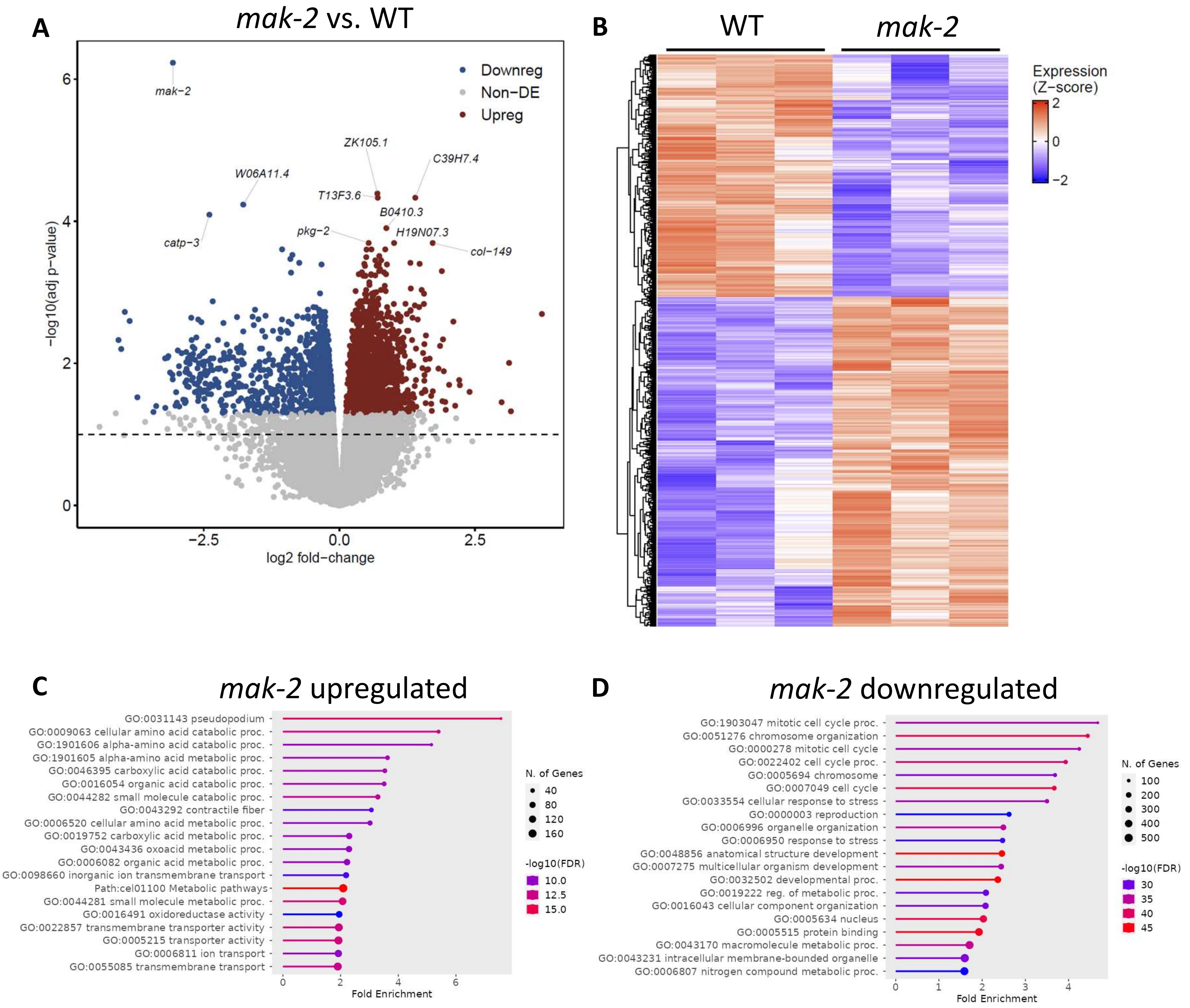

Figure S6. Transcriptional changes in *mak-2* mutants. Gene expression changes in *mak-2* mutants compared to wild-type worms were determined using RNA sequencing. (**A**) Volcano plot comparing gene expression in *mak-2* and wild-type worms. (**B**) Heat map showing differentially expressed genes between *mak-2* and wild-type worms. Gene Ontology (GO) enrichment analysis for genes that are significantly upregulated (**C**) or downregulated (**D**) in *mak-2* deletion mutants compared to wild-type worms.

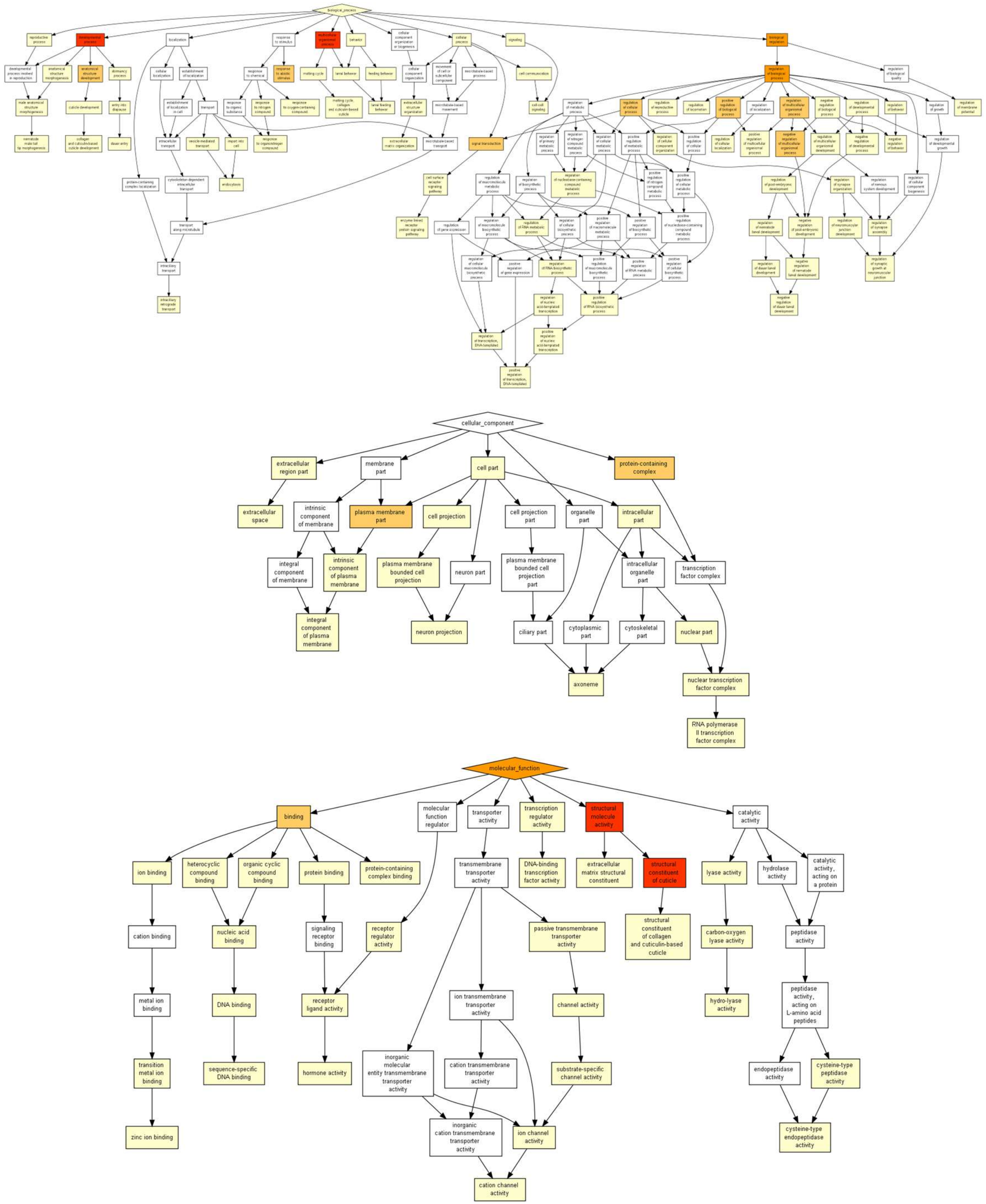

Figure S7. Enrichment analysis for genes upregulated in *isp-1* worms in a *mak-2*-dependent manner. Enrichment analysis was performed using Gene Ontology enRIchment anaLysis and visuaLizAtion tool (GOrilla: https://cbl-gorilla.cs.technion.ac.il/). The genes analyzed were those found to be significantly upregulated in *isp-1* worms but not *isp-1;mak-2* worms and not downregulated in *mak-2* mutants.

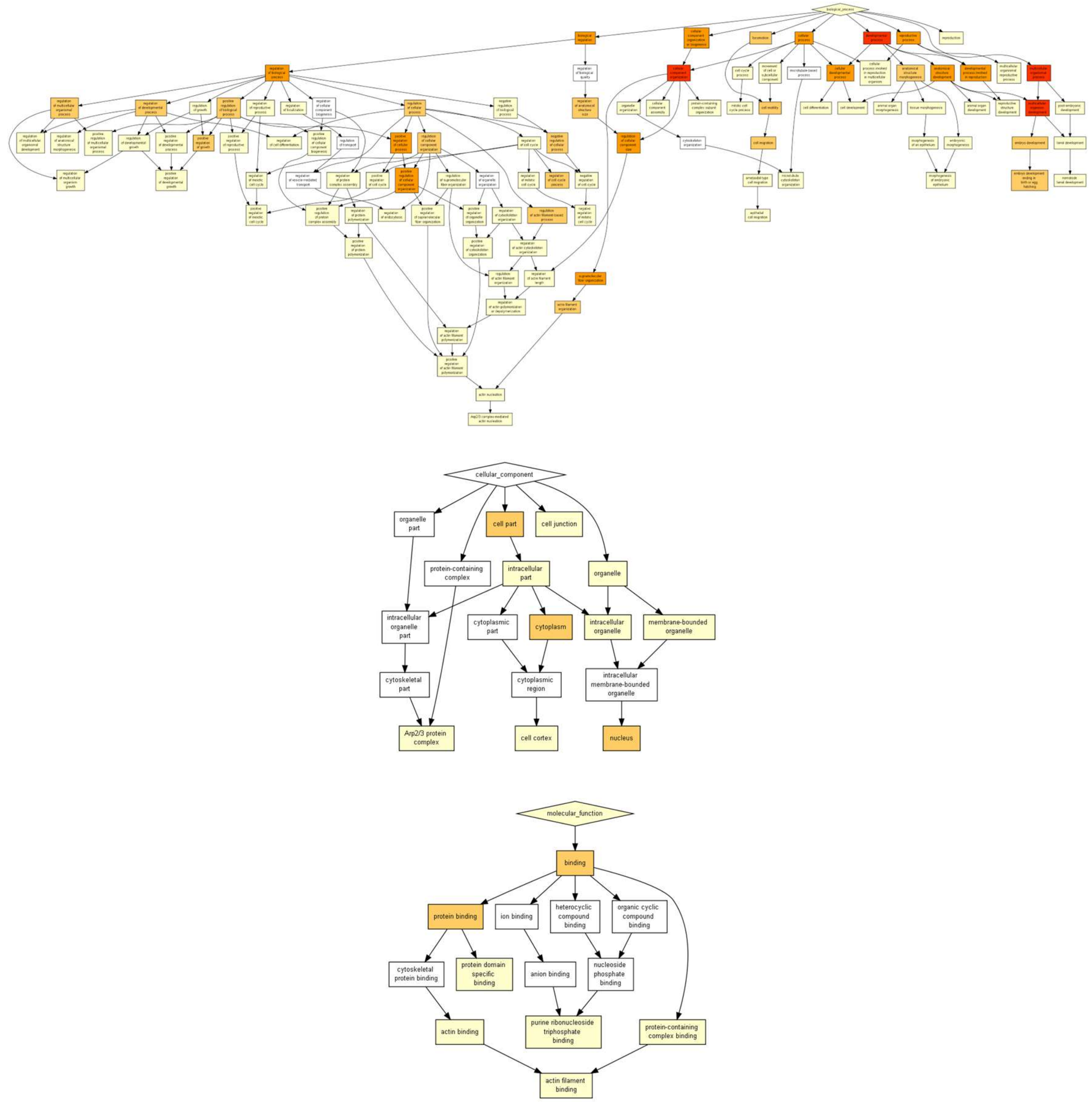

Figure S8. Enrichment analysis for genes downregulated in *isp-1* worms in a *mak-2*-dependent manner. Enrichment analysis was performed using Gene Ontology enRIchment anaLysis and visuaLizAtion tool (GOrilla: https://cbl-gorilla.cs.technion.ac.il/). The genes analyzed were those found to be significantly downregulated in *isp-1* worms but not *isp-1;mak-2* worms and not upregulated in *mak-2* mutants.

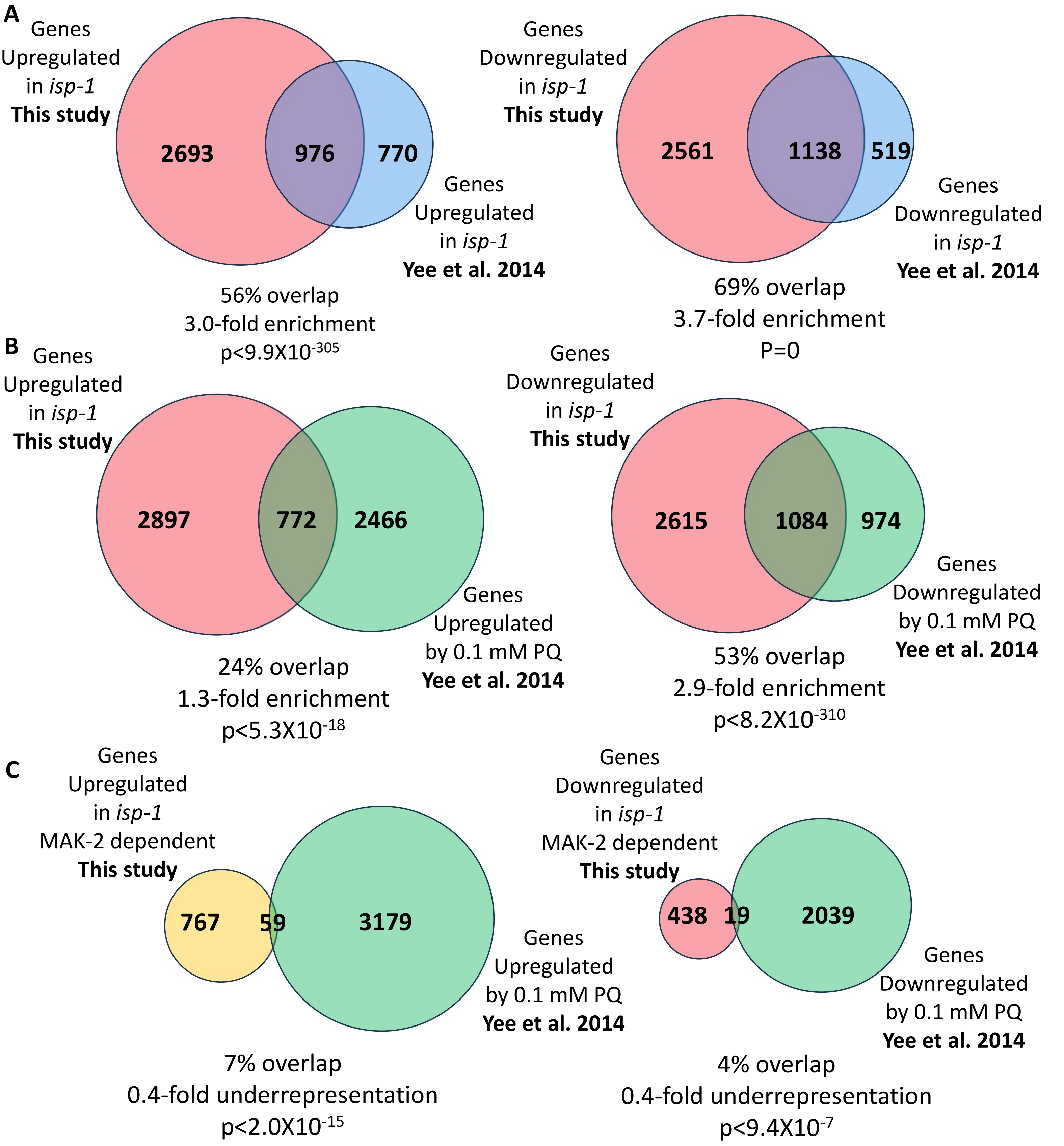

Figure S9. No enrichment of ROS-modulated genes among genes that are differentially expressed in *isp-1* worms in a MAK-2-dependent manner. RNA sequencing results from this study were compared to a published microarray study examining genes expression in worms treated with a lifespan-extending dose of 0.1 mM paraquat (Yee et al., 2014 *Cell*). (**A**) There was a significant degree of overlap between differentially expressed genes in *isp-1* worms from this study and differentially expressed genes in *isp-1* worms from Yee et al. (**B**) There was also a significant degree of overlap between differentially expressed genes in *isp-1* worms from this study and genes differentially expressed after treatment with 0.1 mM paraquat from Yee et al. (**C**) In contrast, genes from this study that were differentially expressed in *isp-1* worms in a MAK-2-dependent manner were underrepresented among genes differentially expressed after treatment with 0.1 mM paraquat from Yee et al. This suggests that the MAK-2-dependent differentially expressed genes in *isp-1* worms are not mediated by elevated ROS.

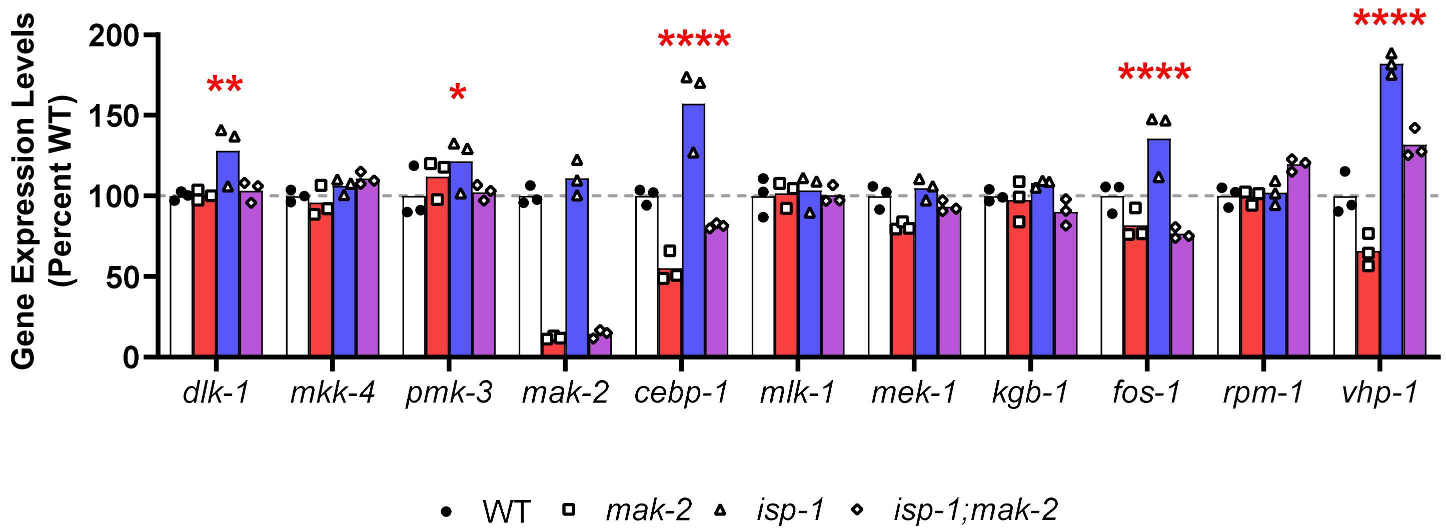

Figure S10. Expression of axonal regeneration pathway components in *isp-1* worms. The expression of axonal regeneration pathway component genes was measured by analyzing data from RNA sequencing. The levels of *mak-2* were unaffected in *isp-1* worms. *isp-1* worms exhibited a small but significant increase in the levels of *dlk-1, pmk-3, cebp-1, fos-1* and *vhp-1.* Statistical significance was determined using a two-way ANOVA with Dunnett's multiple comparisons test. *p<0.05, **p<0.01, ****p<0.0001.

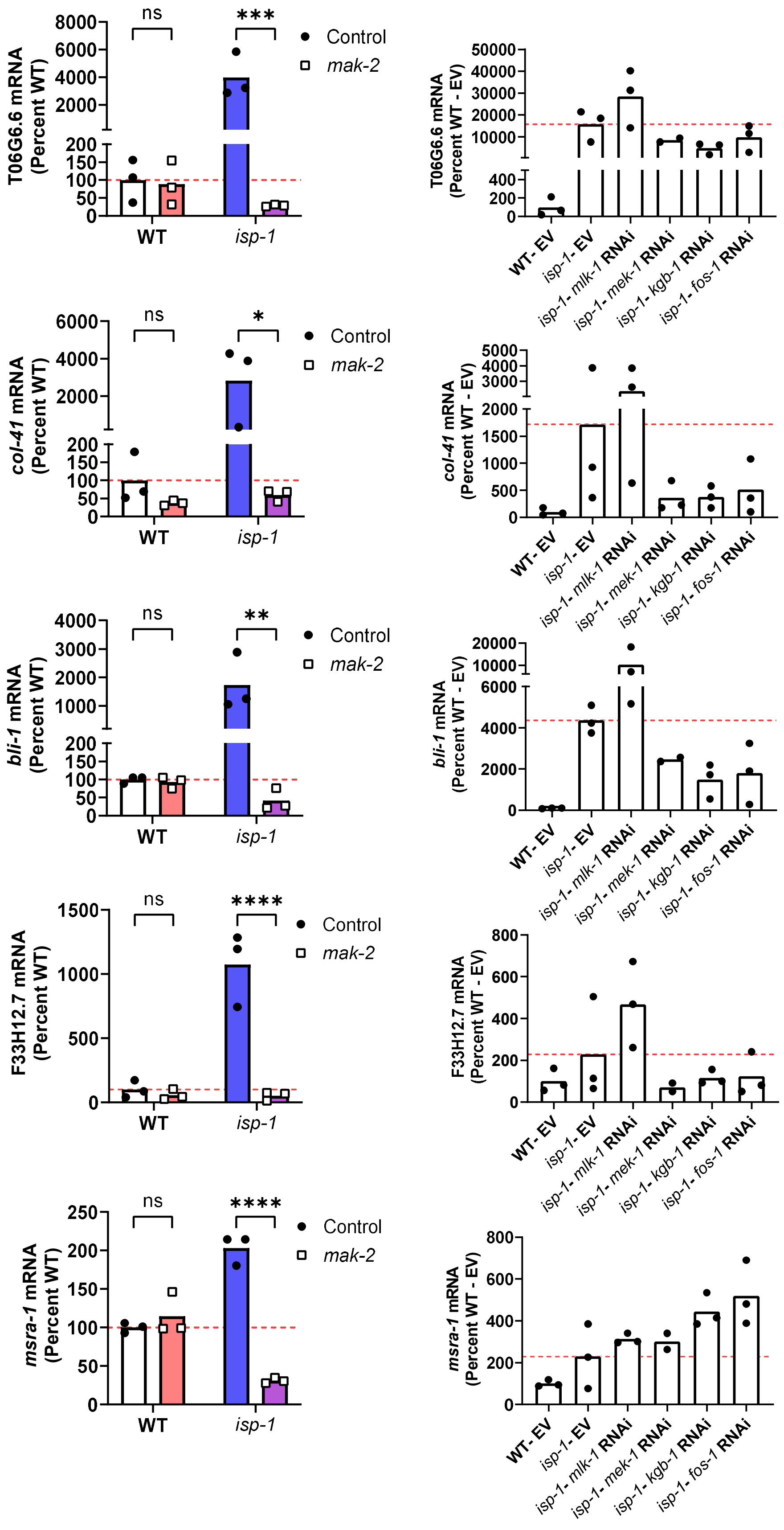

Figure S11. Expression of genes that are upregulated in *isp-1* worms in a MAK-2-dependent manner is not significantly decreased by disruption of genes in the MLK-1/MEK-1/KGB-1/FOS-1 pathway. The left column shows the expression of genes that are upregulated in *isp-1* worms in a MAK-2-dependent manner in wild-type, *mak-2, isp-1* and *isp-1;mak-2* worms. Data was obtained from the RNA-seq results. In each case, the gene is upregulated in *isp-1* worms and the expression is reduced by disruption of *mak-2.* The left column exhibits the expression of these same genes in worms treated with RNAi targeting genes in the MLK-1/MEK-1/KGB-1/FOS-1 pathway. Although a trend towards decrease was observed in some cases, none of the RNAi treatments resulted in a significant decrease in expression. This suggests that MAK-2 modulates the expression of these genes independent of the MLK-1/MEK-1/KGB-1/FOS-1 pathway. Statistical significance was assessed using a two-way ANOVA with Šidák's multiple comparisons test in the left column. *p<0.05, **p<0.01, ***p<0.001, ****p<0.0001.

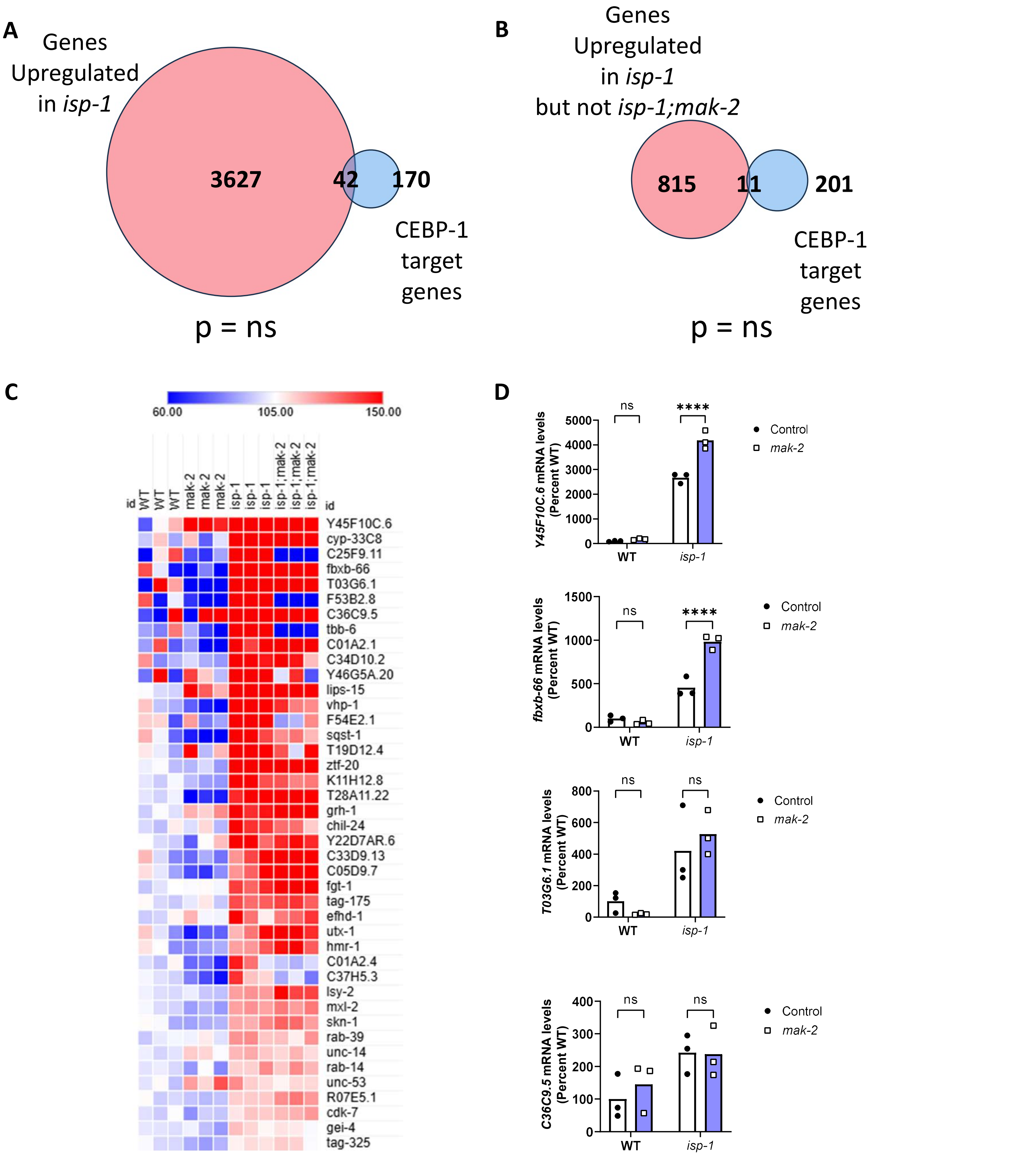

Figure S12. Upregulation of CEBP-1 target genes in *isp-1* mutants is not dependent on *mak-2.* (**A**) Genes upregulated in *isp-1* worms do not show a significant enrichment of CEBP-1 target genes. (**B**) Genes that are upregulated in *isp-1* worms in a *mak-2*-dependent manner do not show a significant enrichment of CEBP-1 target genes. (**C**) The CEBP-1 target genes that are upregulated in *isp-1* worms are mostly also upregulated in *isp-1;mak-2* worms. This suggests that the upregulation of these CEBP-1 target genes in *isp-1* worms is not mediated by MAK-2. (**D**) Examples of CEBP-1 target genes that are upregulated in *isp-1* worms showing a lack of dependence on *mak-2.* Statistical significance was assessed using a two-way ANOVA with Šidák's multiple comparisons test. ns = not significant, ****p<0.0001.

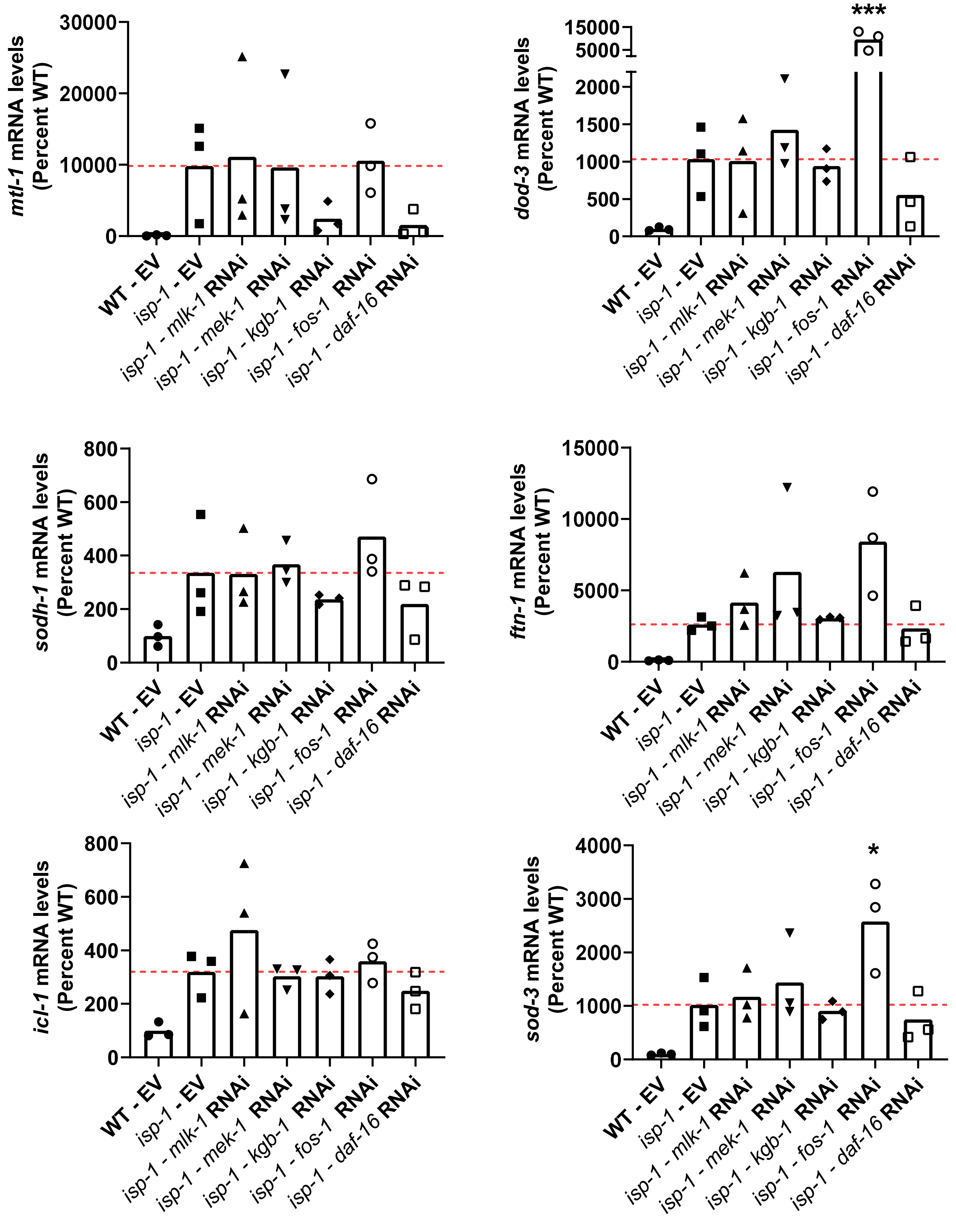

Figure S13. Upregulation of DAF-16 target genes in *isp-1* mutants is not dependent on MLK-1/MEK-1/KGB-1 signaling pathway. The expression of DAF-16 target genes was measured using quantitative RT-PCR after treating *isp-1* worms with RNAi targeting *mlk-1, mek-1, kgb-1* or *fos-1. daf-16* RNAi was included as a control. All of the DAF-16 target genes exhibited a trend towards increase in *isp-1* worms compared to wild-type worms. While *daf-16* RNAi appeared to decrease the expression levels of the DAF-16 target genes, RNAi targeting components of the MLK-1/MEK-1/KGB-1 signaling pathway had minimal effect on the expression of DAF-16 target genes. *fos-1* RNAi significantly increased the expression of *dod-3* and *sod-3.* Three biological replicates were performed. Statistical significance was assessed using a one-way ANOVA with Dunnett's multiple comparisons test. All groups were compared to the *isp-1* – EV group. ns = not significant. *p<0.05, ***p<0.001.

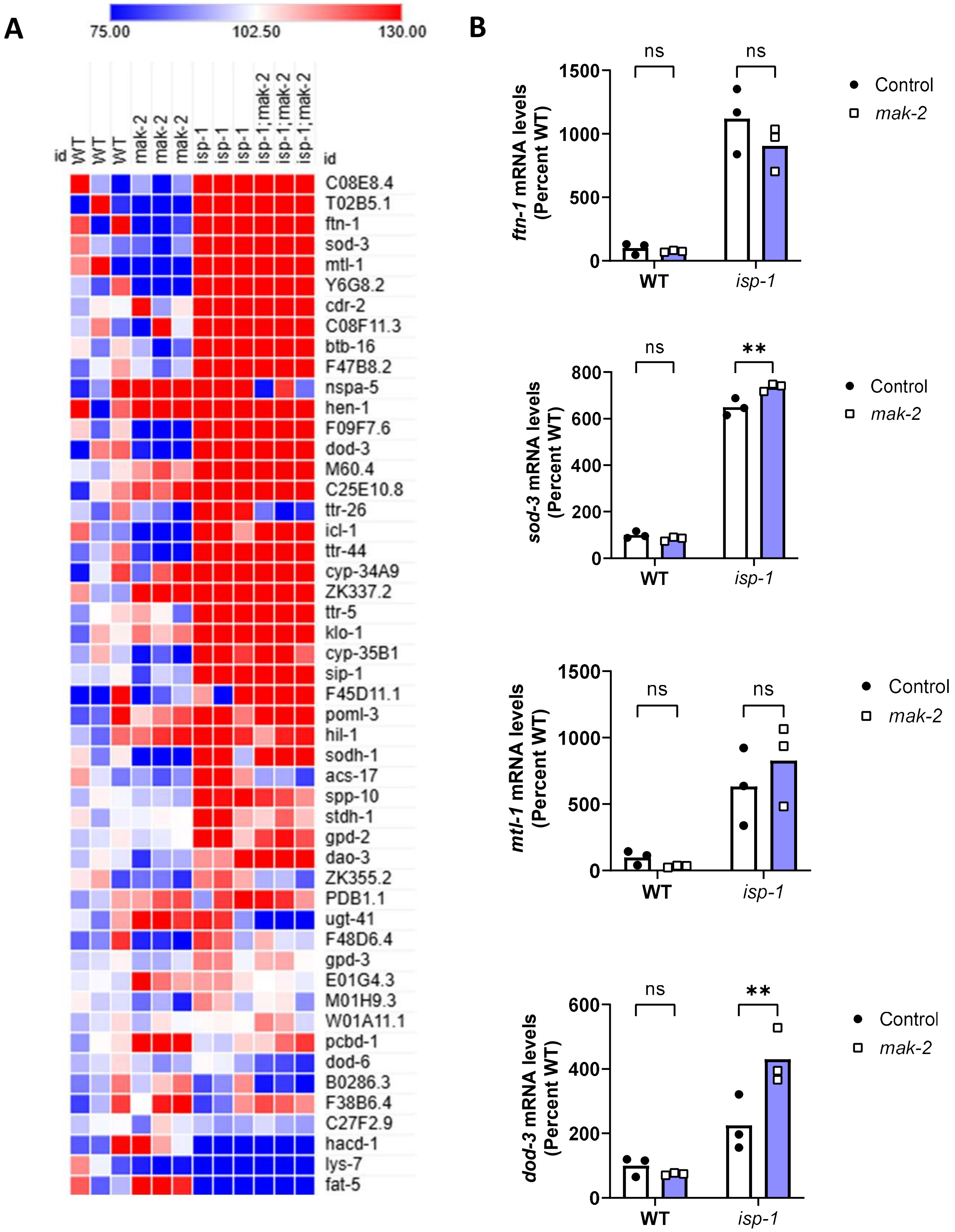

Figure S14. Upregulation of DAF-16 target genes in *isp-1* mutants is not dependent on *mak-2.* The expression of the top 50 consensus DAF-16 target genes from Tepper et al. *Cell* 2013 was examined in wild-type, *mak-2, isp-1* and *isp-1;mak-2* worms. (**A**) The majority of the DAF-16 target genes are significantly upregulated in *isp-1* worms. These target genes are also significantly upregulated in *isp-1;mak-2* mutants. This indicates that *mak-2* is not required for the upregulation of DAF-16 target genes in *isp-1* worms. (**B**) The upregulation of example DAF-16 target genes *ftn-1, sod-3, mtl-1* and *dod-3* in *isp-1* worms is not decreased by the disruption of *mak-2.* Statistical significance was assessed using a two-way ANOVA with Šidák's multiple comparisons test. ns = not significant, $**p<0.01$.

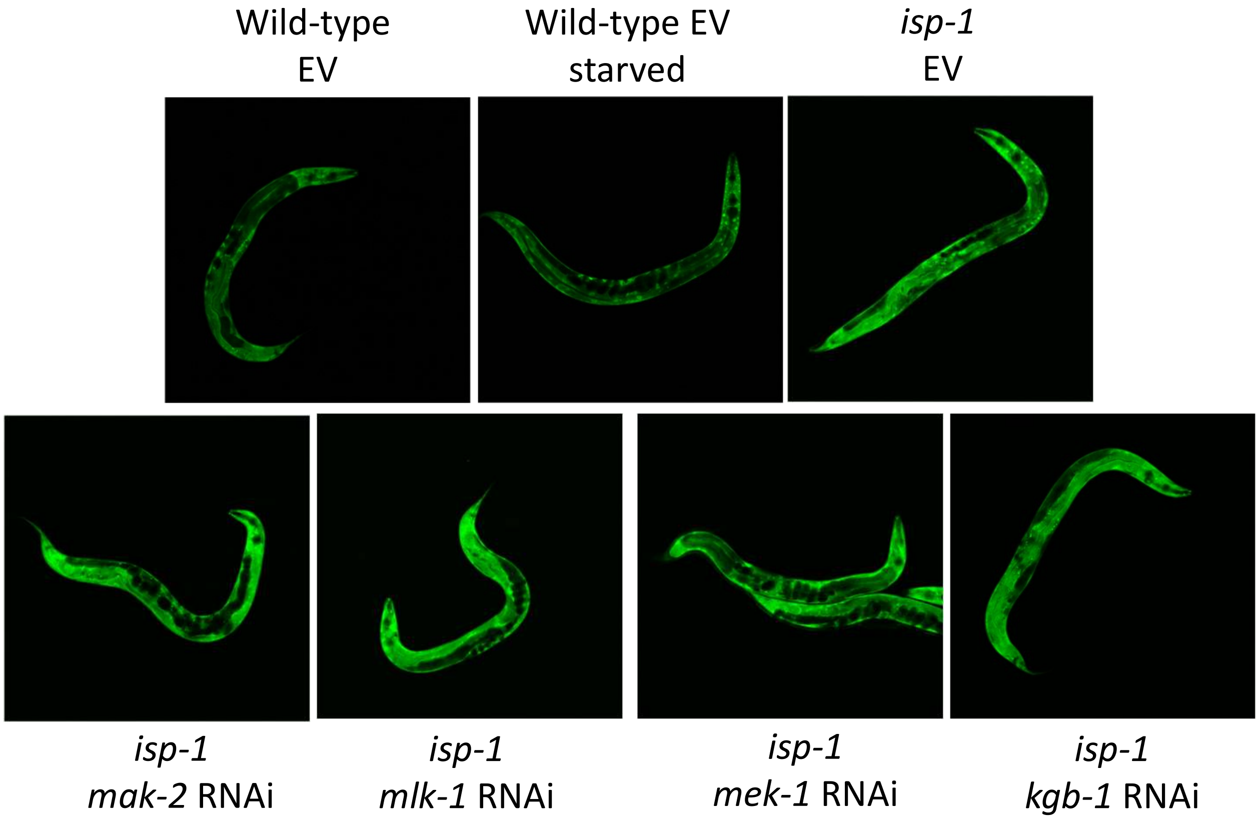

Figure S15. Nuclear localization of DAF-16 is not increased by disruption of genes in the MLK-1/MEK-1/KGB-1 pathway. The nuclear localization of DAF-16 was examined using *zIs356 [daf-16p:daf-16::GFP]* worms. In a wild-type background, DAF-16::GFP is completely cytoplasmic under normal conditions. When these worms were starved, DAF-16::GFP was found to go to the nucleus. In *isp-1;zIs356 [daf-16p:daf-16::GFP]* worms, DAF-16::GFP is mostly cytoplasmic with some DAF-16::GFP moving to the nucleus. Treating *isp-1;zIs356 [daf-16p:daf-16::GFP]* worms with RNAi targeting *mak-2, mlk-1, mek-1* or *kgb-1* did not increase nuclear localization of DAF-16::GFP. Note that since nuclear localization of DAF-16::GFP is minimal in *isp-1;zIs356 [daf-16p:daf-16::GFP]* worms, it was not possible to assess whether RNAi knockdown of these genes diminished nuclear localization of DAF-16.

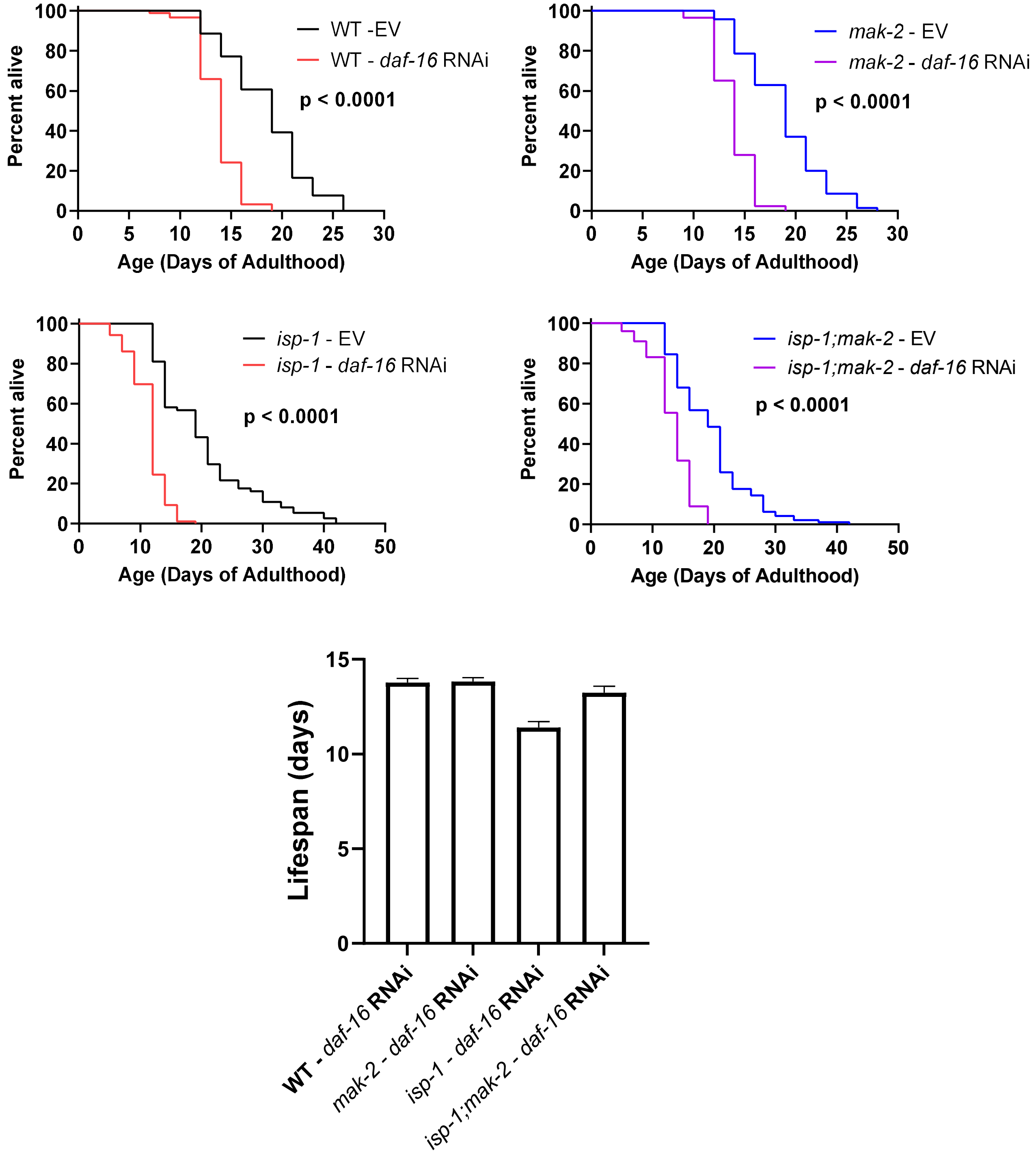

Figure S16. Disruption of DAF-16 decreases lifespan similarly in *isp-1* and *isp-1;mak-2* mutants. To examine how the MAK-2 and DAF-16 pathways interact to determine the lifespan of *isp-1* worms, we knocked down *daf-16* in *isp-1* and *isp-1;mak-2* worms and measured lifespan. We included wild-type and *mak-2* worms as controls. In every case, we found that *daf-16* RNAi markedly decreased lifespan. The lifespan of all four strains was similar when treated with *daf-16* RNAi. There was no additive effect of disrupting *mak-2* and *daf-16.* Statistical significance for the survival plots was determined using the log-rank test.

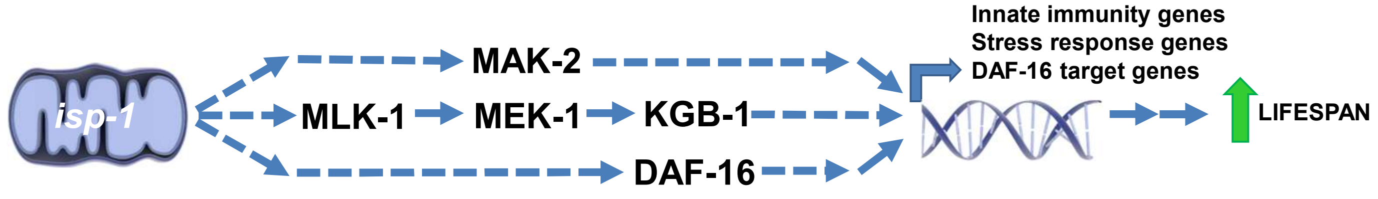

Figure S17. Model for contribution of different signaling pathways to *isp-1* lifespan. MAK-2, MLK-1, MEK-1, KGB-1 and DAF-16 are all required for the extended longevity of *isp-1* worms. Neither the MAK-2 pathway nor the MLK-1/MEK-1/KGB-1 pathway are required for the activation of DAF-16 target genes in *isp-1* worms. *isp-1* worms do not show an enrichment of CEBP-1 target genes and those CEBP-1 target genes that are upregulated in *isp-1* worms are largely independent of MAK-2. Our data suggests that MAK-2, DAF-16 and the MLK-1/MEK-1/KGB-1 pathway are acting independently to promote longevity in *isp-1* worms. Genes upregulated in *isp-1* worms include innate immunity genes, stress response genes and DAF-16 target genes.
